# Upcycling Brewer’s Spent Grain and Barley Rootlets by Partial Substitution of Pea Protein Isolate in Extruded High Moisture Meat Analogues

**DOI:** 10.3390/foods15081327

**Published:** 2026-04-10

**Authors:** Ivana Salvatore, Robin Betschart, Claudio Beretta, Maria Rudel, Evelyn Kirchsteiger-Meier, Corinna Bolliger, Matthias Stucki, Nadina Müller

**Affiliations:** Department of Life Sciences and Facility Management, Zurich University of Applied Science (ZHAW), Einsiedlerstrasse 35, 8820 Wädenswil, Switzerland; ivana.salvatore@gmx.ch (I.S.); robinbetschart@icloud.com (R.B.); beet@zhaw.ch (C.B.); meryrudel@gmail.com (M.R.); meev@zhaw.ch (E.K.-M.); boir@zhaw.ch (C.B.); stck@zhaw.ch (M.S.)

**Keywords:** beer brewing by-products, brewer’s spent grain, barley rootlets, high-moisture meat analogues, high-moisture extrusion, side stream valorisation, novel food status, sustainability, pea protein isolate

## Abstract

This study evaluated how a partial substitution of pea protein isolate (PPI) with brewer’s spent grain (BSG) or barley rootlets (BRs) affects high-moisture meat analogues (HMMAs). PPI was substituted with 10% and 20% with BSG or BRs, respectively. Extrudates were produced on a co-rotating twin-screw extruder at maximum temperatures of 140 °C and 160 °C. Extrudates were assessed for colour, moisture, firmness and fibre morphology. Furthermore, the technofunctional and nutritional properties of the raw materials were determined. Extrudates with BSG produced the darkest colour, whereas PPI and BR formulations exhibited the lightest. A stronger reddish tint was observed at 160 °C, while the colour within the yellow–blue spectrum was largely temperature-independent. Firmness was generally higher at 160 °C, consistent with lower end-product moisture. Side stream addition lowered protein content and weakened fibre formation, with the effect most pronounced for BRs. Overall, formulation was the dominant factor influencing lightness, while temperature modestly increased redness and firmness. Preliminary sensory evaluation supported these trends. Extrudates produced at 140 °C were perceived as having a more fibrous structure. Higher substitution levels resulted in a weaker, more crumbly texture. With respect to the environmental assessment, a 20% replacement of PPI with BRs or BSG reduced overall environmental impacts by up to 19% and climate impacts by up to 16%. With regard to the novel food status, the EU Novel Food Status Catalogue classifies BSG as not novel, whereas BRs are not novel only when used in food supplements. Any other food uses, other than as, or in, food supplements, might considered to be novel and consequently might need to be authorised under the novel food regulation framework prior to market placement.

## 1. Introduction

Large volumes of potentially edible material are lost during agri-food processing, so strategies that keep nutrients in the human food chain through upcycling and circular design are needed [1]. Side streams from the brewing and malting sector are particularly interesting because they are generated in high volumes and nutritionally promising. They consist mainly of barley rootlets (BRs), the sprouts removed from malt after germination, and brewer’s spent grain (BSG), the solid residue remaining after wort separation [2]. BRs are produced during the malting step and typically account for 3–5% of the malt mass [3]. Worldwide, 140 million tonnes of barley are harvested per year (base year 2023), thereof about 21% are malted, resulting in 0.9–1.5 million tonnes of BRs per year [4,5]. For every 100 L beer, approximately 20 kg BSG are generated [6]. Globally, breweries produce roughly 39 million tonnes of BSG per year, with an estimated production of 3.4 million tonnes in Europe alone. At present, both materials are largely used in low-value applications such as ruminant feed, composting or biogas, yet their composition suggests that they could serve as nutritionally and technologically valuable ingredients for food [3,7].

BRs are dried to relatively low moisture contents after kilning, making them more stable than wet BSG, but they are highly hygroscopic and can reabsorb water during storage [3]. Several studies have explored their inclusion in food products. In particular, their inclusion in bread and bakery products has been successfully investigated [8,9]. From a food safety perspective, BRs pose an elevated risk for mycotoxins, since the sprouting tissue provides favourable conditions for Fusarium and Aspergillus growth [10,11,12].

BSG is the wet solid fraction after lautering and, when fresh, contains 70–80% moisture, which can limit shelf life if not preserved accordingly [2]. In the last decade, BSG has been increasingly explored as a functional ingredient in human food. BSG flours and fractions have been incorporated into a broad range of cereal-based products, including bread, cookies, cereal bars, crackers, extruded snacks, breakfast cereals and pasta, generally increasing dietary fibre and protein content while moderating caloric density [13,14,15,16]. Da Silva et al. demonstrated that replacing up to 15% soy protein with BSG in high-moisture meat analogues (HMMAs) increased fibre content while maintaining overall texture [17]. Another study, by Fan et al., included only 0.3–6% BSG, demonstrating its functionality as a structural enhancer in faba bean protein-based HMMAs [18]. A recent review concluded that BSG can simultaneously improve nutritional quality, provide natural antioxidants and contribute to texture stabilisation when appropriately processed, especially via extrusion and fermentation [19].

Taken together, BSG and BRs represent complementary side streams from brewing and malting that are abundant, nutritionally attractive and technologically promising. Using these side streams as structural and nutritional ingredients in HMMAs offers an opportunity to increase resource efficiency and reduce reliance on purified protein isolates. However, this approach also raises important questions regarding processability and safety that require systematic investigation. The market for plant-based meat analogues (PBMAs) has grown rapidly, but often relies on purified protein isolates from, for example, pea or soy, which come with cost, supply and sustainability drawbacks linked to fractionation. This results in large amounts of new side streams and long supply chains [20]. Substituting part of these isolates with locally available side streams can potentially reduce dependence on raw materials while creating value for manufacturers. PBMAs already represent a meaningful market, so even partial substitution would have system-level effects. Retail sales of plant-based meat and seafood were USD 6.1 billion globally in 2024 [21], and CHF 86.8 million for meat substitutes in Switzerland in 2023 [22].

High-moisture extrusion cooking (HMEC) is the dominant top-down technology used to make HMMAs with anisotropic, fibrous textures. HMEC converts a raw material blend, primarily plant protein concentrates or isolates, into fibrous, non-expanded extrudates. These extrudates have a moisture content of at least 40% [23,24]. During extrusion, the material passes through four processing zones (feeding, mixing, melting and cooling), which drive material transformations. In the melt zone, strong shear and temperatures of 130–180 °C promote component melting (plasticisation) and markedly raise barrel pressure. Under these conditions, molecules lose their original three-dimensional structure and dissociate into subunits as chains unfold. Heat-labile non-covalent and covalent disulfide bonds also break [23,25]. Immediate cooling via a cooling die after the melt zone is essential because sudden pressure release at die exit can vaporise residual water. The resulting steam may impair or destroy the HMEC fibrous structure, leading to an undesired expansion. Premature in-barrel cooling is also problematic: at high screw speeds, freshly solidified material can be re-disrupted, undermining controlled structure formation [23,26]. In the cooling die, laminar flow and a temperature gradient arise because the core cools more slowly than the wall, resulting in a velocity gradient that produces the characteristic V-shaped structure inside the extrudate [27,28].

The macronutrients starch, proteins, lipids and dietary fibre influence extrudate properties in different ways. Plant proteins are the main component of meat substitutes, the most commonly currently used being soy proteins, wheat gluten and proteins from selected legumes, owing to their ability to closely mimic meat-like texture and structure. Empirical studies suggest that concentrates or isolates with at least 50–90% protein in the feed are required, because lower protein contents markedly impair the formation of a fibre-like structure in the extrudate [20,26,29].

Increasing protein content generally strengthens the thermogelling effect and enhances chemical bonding, both of which support fibrous structure formation. Lubricity in the extruder is reduced, significantly improving firmness, brittleness and the rheological properties of extruded meat analogues [30,31,32]. The type and amount of protein used strongly affect the extent of fibre formation [20,26,29].

Starch can be added in small quantities to optimise extrudate structure in meat substitutes due to the ability of polysaccharides to combine with exposed reactive sites on protein chains and enhance protein network formation. Adding starch can improve water holding, thickening and cohesion, but excessive addition can harm cutting force, texture and elasticity [30].

Insoluble fibre in low-moisture meat analogues (LMMAs) increased melt viscosity and reduced swelling, yielding denser, lower-moisture extrudates. In HMMAs, replacing 10–20% of soy protein isolate with insoluble fibre promoted a pronounced fibrous structure at macro and micro scales, and with higher fibre levels, tensile strength rose while elasticity and hardness decreased [33].

Fats and oils exert a significant influence on extrusion cooking by acting as lubricants, reducing friction both between particles in the blend and between screw and barrel surfaces and the melt. Adding lipids can enhance tenderness, juiciness and flavour in plant-based meat substitutes [34]. It was observed that phospholipids in particular can exert a greater impact on protein network development than extrusion temperature or moisture content [30].

Besides formulation, process parameters have a major impact on fibre formation during high-moisture extrusion cooking (HMEC). Several effects of key parameters such as temperature, moisture, and screw speed are well-established in the literature for protein texturisation: Barrel temperature is a key parameter for protein texturisation, with the optimal range varying depending on protein source and moisture content [35]. For pea protein, extrusion below 120 °C produced soft, doughy, non-fibrous extrudates, whereas higher temperatures yielded multilayered structures, with predominantly longitudinal fibres observed at 160 °C [36]. Elevated extrusion temperatures can also improve protein–polysaccharide compatibility, reducing microstructural interfaces between phases. While this can increase product strength, it may also decrease the degree of visible texturisation [29].

Moisture content is equally critical, as it increases the mobility of reactive protein molecules and facilitates chain stretching and alignment, thereby promoting cross-linking reactions [37]. Higher moisture levels are also associated with a lower temperature rise in the material, as friction and mechanical energy input are minimised and dissipated as heat [38]. Moisture contents in HMMAs range from 40% to 80%, while optimal fibre structure was achieved at moisture contents of 55–60% [39,40,41]. Excess water, by contrast, yields products with a spongy consistency and reduced fibre formation [42].

Lastly, higher screw speed increases barrel heat, lowers melt viscosity and causes a die pressure drop. Higher screw speed intensifies shear, dispersing the carbohydrate-rich dispersed phase within the continuous protein phase and yielding a finer, more distinct fibrous structure. It also promotes chain stretching and alignment along laminar flow, forming parallel layers, but its effect is smaller than that of barrel temperature and water feed [23,25,31,43].

This study aimed to (a) quantify how 10% and 20% substitution of PPI with BSG or BRs affects colour, moisture, firmness, and macro fibrous morphology of HMMAs at two peak barrel temperatures (140/160 °C); (b) characterise technofunctional and safety properties of BSG and BRs; (c) evaluate environmental implications via LCA; and (d) clarify the EU/Swiss novel food status. The novelty of this study lies firstly in demonstrating the feasibility of incorporating brewers’ spent grain (BSG) into high-moisture meat analogues (HMMAs) at higher inclusion levels than previously reported, in introducing BRs as an alternative ingredient at the same, substantial inclusion levels. Secondly, this work provides a comprehensive assessment beyond technological feasibility, also covering nutritional properties, food regulatory considerations, and environmental impacts, thereby offering an integrated perspective on the potential of BSG- and BR-based HMMA in sustainable food systems.

## 2. Materials and Methods

### 2.1. Characterisation of Raw Material

Mill-dried brewer’s spent grain (BSG) was obtained in 2024 by Blattmann Schweiz AG (Art. No. 17001, Wädenswil, Switzerland). Barley rootlets (BRs) were obtained in 2024 from Schweizer Mälzerei AG (Möriken-Wildegg, Switzerland) and were milled by impact roller mill SR300 (Retsch, Haan, Germany) using a 200 μm sieve. Pea protein isolate (PPI) (“Erbsenproteinisolat 80%”) was bought from Ceresal (Mannheim, Germany) and not further milled.

For PPI, the nutritional information was available from the manufacturer’s specifications. For BSG and BRs, the fat concentration was measured using a rapid NMR Fat Analyzer ORACLE (CEM Corporation, Matthews, NC, USA). The protein contents of BSG and BRs were quantified by LaborVeritas (Zurich, Switzerland), and the contents of soluble and insoluble dietary fibre were determined by the lab Eurofins (Schönenwerd, Switzerland). To allow for a risk assessment of the side streams, the contents of mycotoxins (screening of Aflatoxin B1, B2, G1, G2, Deoxynivalenol (DON), Nivalenol, Ochratoxin A, Zearalenone (ZON), T-2-Toxin, HT-2-Toxin, Fumonisin B1 and B2, all results in μg/kg, measurement method LC-MS/MS), NDMA (N-Nitrosodimethylamine) (only relevant for BRs [44]), *Bacillus cereus* (only relevant for BSG [45], ISO 7932 [46]), aerobic mesophilic bacteria count (ISO 4833-1, [47]), yeasts and moulds (ISO 21527-2 [48]) were determined by LaborVeritas (Zurich, Switzerland). All externally performed analyses were conducted by accredited labs and using one sample. Sampling was done by mixing portions from all obtained material to ensure representative results.

#### 2.1.1. Water- and Oil-Holding Capacity (WHC/OHC)

WHC and OHC were determined following Raikos et al. [49] with minor modifications. First, 1.0 g of sample was combined with 12.0 g of either deionised water or rapeseed oil (Florin AG, Muttenz, Switzerland) in 15 mL centrifuge tubes, vortexed for 1 min and centrifuged at 845 rcf for 30 min (Centrifuge 5810, Vaudaux-Eppendorf AG, Schönenbuch, Switzerland). In contrast to Raikos et al., a 12 g liquid addition (instead of 10 mL), 15 mL centrifuge tubes (instead of 50 mL), a defined vortex time of 1 min, and a lower centrifugation force (845 rcf vs. 3148 g) were applied. After decanting the supernatant, the residue was weighed to quantify retained water or oil. WHC and OHC were calculated as described in Equation (1). WHC and OHC analyses were performed in triplicate.

(1) $$WHC/OHC\;[\%]=\frac{\left(m_{Water/Oil}-m_{Supernatant}\right)}{m_{Sample}}\times 100$$

$$m_{Water/Oil}\;[g]:Mass\;of\;water\;or\;oil$$

$$m_{Supernatant}\;[g]:Mass\;of\;supernatant\;after\;centrifugation$$

$$m_{Sample}\;[g]:Mass\;of\;sample$$

#### 2.1.2. Water Absorption (WA) Potential

WA was assessed using the method of Lin et al. [50] with minor modifications. First, 4.5 g of sample was combined with 30 g of water in a 50 mL tube, shaken manually for 1 min and refrigerated at 4 °C for ≥12 h. The resulting slurry was poured onto a porcelain funnel fitted with a MN 640 w filter (MACHEREY-NAGEL, Düren, Germany) with pore size 7–12 μm. The retentate was weighed and then dried at 40 °C (to avoid thermal transitions of sample components) until constant mass was achieved (mass change < 0.01 g over 1 h) to quantify water uptake. WA, expressed on a dry-matter basis, was calculated according to Equation (2). Water absorption analyses were performed in triplicate.

(2) $$WA\;[\%\;d.m.]=\frac{\left(m_{wet}-m_{dry}\right)}{m_{dry}}\times 100$$

$$m_{wet}\;[g]:Mass\;of\;wet\;slurry$$

$$m_{dry}\;[g]:Mass\;of\;dried\;sample$$

#### 2.1.3. pH Value

A 10 g sample was mixed with 100 g distilled water and allowed to stand for 5 min. Measurements were performed with a pH metre (CG 837, Schott, Mainz, Germany), calibrated with standard buffer solutions at pH 4.00, 7.00, and 10.00, at room temperature (20–22 °C). Immediately before measurement, the mixture was remixed, and pH was measured directly in the slurry without filtration or decantation. For each sample, six determinations were conducted.

#### 2.1.4. Particle Size

Particle size distributions (PSDs) were measured with a Camsizer X2 equipped with the X-Jet dry-dispersion module (Microtrac Retsch GmbH, Haan, Germany) at 3 bar compressed air. Acquisition was triggered at 70% nominal area density. Images were captured by the CCD-Basic and CCD-Zoom cameras. Each run was stopped after 40,000 images or when low-density images reached 0.01%. Per material, six measurements were performed. Data were evaluated as volume-based distributions using the Camsizer X2 software (v6.10.4.1221). Distributions were characterised by 10% particle size (x_10,3_), median particle size (x_50,3_) and 90% particle size (x_90,3_).

#### 2.1.5. Moisture

Moisture content was determined using a halogen moisture analyser (HS153, Mettler Toledo, Greifensee, Switzerland) and 1.00 g of sample. The “gentle drying” programme was used with a ramp time of 3 min and a drying temperature of 105 °C. Each raw material was analysed in triplicate.

### 2.2. Production of HMMA Extrudates

A lab-scale, co-rotating, intermeshing twin-screw extruder (ZE18, Three-Tec, Seon, Switzerland) was used. The heated barrel is divided into eight elements, including seven zones that can be heated and cooled independently (Z1–Z7). Zone 0 was cooled using a recirculating chiller set to 15 °C (HRC 2 B S000, IKA, Staufen im Breisgau, Germany). A cooling die (H 10 mm × W 30 mm × L 280 mm) was mounted at the outlet and supplied by a recirculating chiller set to 5 °C (HRC 2 B S000, IKA, Staufen im Breisgau, Germany). A breaker plate was installed between the barrel outlet and the cooling die inlet. For the water feed, a dosing pump (DP MZR-7205, Three-Tec, Seon, Switzerland) with an inline filter (F-MI3-T-s-e1 25, HNP Mikrosysteme, Schwerin, Germany) was used. Two identical screw configurations were mounted, each comprising 32 elements drawn from nine screw element types to suit HMMA production. The sequence of elements is shown in Figure S1 in Supplementary Material S1. Dry raw materials were fed using a twin-screw feeder (ZD 12 FB-C-1M-DN200, Three-Tec, Seon, Switzerland) driven by a motor (SK71S/4TF, Getriebebau NORD AG, Arnegg, Switzerland).

Two batches per formulation were produced on consecutive days. To ensure comparability across samples, production, cooling, packaging and analysis followed an identical procedure. The samples’ process parameters and formulation compositions are shown in Table 1. The raw materials were PPI, mill-dried BSG and milled BRs. Additionally, a powdered beef flavour (“Rindfleisch Aromapulver natürlich”, Günter Aroma, Beinwil am See, Switzerland) was added to the mixtures at 15 g/kg. Per the recipe, 1 kg of product was produced. The dry ingredients for each formulation were weighed and mixed thoroughly before they were filled in the feeder. Dry raw material was fed into zone 0 of the extruder at 1100 g/h through a scale dispenser (TT/S FTC, Three-Tec, Seon, Switzerland). Water was added between zone 1 and zone 2 of the extruder. The water feed was set to obtain a target moisture of 65% in the end-product (based on [25] and preliminary trials) and when necessary, adapted to maintain smooth and even processing. This resulted in a non-identical feed setting for the different recipes and consequently, slightly varying end-product moistures. Furthermore, even though extrusion is a closed system, moisture variations occur due to steam leaks between the barrel segments and evaporation from the warm extrudates further contributed to variations in end-product moisture content. The extrusion screw speed was set to 500 rpm.

The seven heating zones (Z1–Z7) of the extruder were set to Z1 50 °C, Z2 65 °C, Z3 80 °C, Z4 100 °C, Z5 120 °C, Z6 140° or 160 °C, Z7 120 °C. To assess the effect of a higher maximal extrusion temperature, the heating zone Z6 was set at 140 °C and at 160 °C for the performed trials, resulting in two different samples per formulation, identifiable in the sample code by _140 and _160. One formulation (BR2) was only extrudable at 140 °C and could not be processed at 160 °C due to an unstable process.

For each formulation, strands of approximately 20–30 cm were cut for analyses. Samples were covered with cling film (PVC, Cofresco Frischhalteprodukte GmbH & Co. KG, Minden, Germany) to prevent surface drying and first cooled to room temperature. Colour and moisture were measured at room temperature on these strands. The remaining strands were packed in plastic bags (PA/PE, Pistor, Rothenburg, Switzerland) and cooled; the bags were sealed once products reached 3 °C. Macroscopic images were taken on these 3–cooled (never-frozen) strands. Only samples allocated for texture analysis were frozen at −20 °C until analysis.

### 2.3. Characterisation of HMMA Extrudates

#### 2.3.1. Colour

Colour was measured immediately after extrusion using a chromameter (CR-400, Konica Minolta, Tokyo, Japan). To ensure consistent lighting conditions, all measurements were taken with identical lighting conditions. Six measurements were performed per batch, resulting in twelve data points per sample. Colour was recorded in the CIELAB colour space, which covers the full range of human colour perception and enables precise colour-difference measurements, using L*, a*, and b* values. The a* axis spans green (negative) to red (positive), the b* axis blue (negative) to yellow (positive), and L* indicates lightness (0 = black, 100 = white) [51]. In addition, the Delta E values were calculated as an indicator of whether a colour difference is perceptible to the human eye. According to the literature, ΔE < 1.5 indicates slight differences, 1.5–3 noticeable differences, and >3 very noticeable differences. ΔE was calculated using the Formula (3) below [51]. In addition to the samples, the colour of the raw materials (BSG, BRs and PPI) was analysed identically in six-fold.

(3) $$\Delta E=\sqrt{\left(\Delta {L^{*}}^{2}+\Delta {a^{*}}^{2}+\Delta {b^{*}}^{2}\right)}$$

#### 2.3.2. Firmness

The extrudates were removed from the freezer the day before analysis and thawed overnight in a refrigerator at 3 °C. At the time of analysis, all products had a core temperature of 20 °C, measured with a thermometer (Testo 925, Testo AG, Titisee-Neustadt, Germany). Firmness was measured in sextuplicate per batch, resulting in twelve measurements per formulation. Measurement was performed on a texture analyser (TA.XT plus, Stable Micro Systems, Godalming, UK) equipped with a 50 kg load cell and a Warner-Bratzler blade following a method recommended by the manufacturer. Samples matched the die dimensions (10 mm height × 30 mm width) and were cut to 90 mm length. Tests were run in displacement-controlled mode with a pre-test speed of 2.00 mm/s, test speed of 2.00 mm/s and return speed of 10.00 mm/s. The target travel distance was 50.00 mm with a trigger force of 20.0 g. The maximum and total cutting forces required to sever the extrudate were recorded.

#### 2.3.3. Macroscopic Analysis

Visual analysis of the extrudates was performed using a digital macroscope (FHD Trend, Tagarno, Horsens, Denmark) equipped with a macro lens (4×, 58 mm, Opteka, Brooklyn, NY, USA). At the start of the examination, a white sheet of paper was placed under the lens to perform white balance; the paper was then used as the background for imaging the samples. Samples were cut into 60 mm pieces and manually torn along the melt flow direction to expose the internal fibre structure. Images were acquired at 30× magnification.

#### 2.3.4. Final Composition

Moisture content was determined immediately after extrusion using a halogen moisture analyser (HS153, Mettler Toledo, Greifensee, Switzerland). Approximately 3 g of extrudate were cut into small cubes (≈2 mm), weighed and evenly distributed on the sample pan. The “Standard drying” programme was run at 105 °C with a 3 min ramp. For each batch, duplicate measurements were performed, yielding four values per formulation.

The final composition was calculated based on the nutritional specification sheets (PPI and BSG), Waters et al., 2013 [9] for BRs, while considering water loss during extrusion.

### 2.4. Environmental Assessment

In this study, a life cycle assessment (LCA) was conducted to evaluate the environmental performance of valorisation and utilisation scenarios for brewery side streams. The LCA followed the four steps described by ISO 14,040 and 14,044 standards [52,53]. and was performed using the SimaPro software 10.2 (PRé Sustainability, Amersfoort, The Netherlands). Modelling was based on primary data from this study and background data from ecoinvent v3.11 [54]. The impact assessment was carried out using the comprehensive Ecological Scarcity Method that covers 19 environmental impact categories [55]. Each impact is characterised with an eco-factor based on the distance-to-target principle, which compares current emission flows to target limits. Results for the single-score indicator are expressed in eco-points (EPs), allowing for direct comparison across products [54]. Additionally, greenhouse gas emissions using the IPCC 2021 GWP 100 method [56] was computed for the different HMMA scenarios. For each HMMA formulation, the higher process temperature (160 °C) was considered. The variant BR2_140 was the only one not extrudable at 160 °C; thus, the only variant considered at 140 °C temperature. The assessment covered the full life cycle from raw material extraction to disposal (i.e., cradle to grave).

In a first assessment, we quantified the environmental impacts of integrating BSG or BRs into HMMAs for a functional unit of 1 kg of final product ready for use (see Tables S1–S3 in Supplementary Material S2). Although BSG and BRs are by-products, they hold nutritional and economic value. Accordingly, environmental impacts were distributed between the main and side products using economic allocation reflecting relative market values. Of the total environmental impact of the brewery processes, 0.22% was assigned to BSG and 0.02% to BRs, with the remaining 99.76% allocated to the beer (see Table S15 in Supplementary Material S2). The economic allocation was selected as production decisions are primarily driven by economic value rather than physical relationships between co-products.

In a second assessment, we compared valorisation pathways for BSG and BRs with alternative utilisation scenarios (feed, heat and electricity co-generation via incineration and via biogas, composting). For this, a system expansion with substitution approach was applied (see Tables S4–S14 in Supplementary Material S2). The functional unit for the comparison was defined as the valorisation or utilisation of 1 kg of BSG and 1 kg of BRs, enabling consistent comparison across heterogeneous outputs. Each output was related to the quantity of a reference product substituted when side stream-based alternatives enter the market. PPI_160 served as the substitution product for the HMMA. For composting, substitution was based on the equivalent NPK content of mineral fertiliser. For energy of incineration and biogas, the substituted products were the Swiss average electricity mix and natural gas (central or small-scale) for the energy produced. Feed substitution was modelled with a mixture of wheat bran, proti-grain DDGS (Distillers Dried Grains with Solubles) and sunflower cake, providing the same nutrients. DDGS is generated as a by-product of the corn processing stage in ethanol production. Details on system boundaries, reference products, inventory data and allocation procedures are provided in the Supplementary Material S2 [2,3,13,57,58,59,60,61,62,63,64,65,66,67,68,69,70,71,72,73,74,75,76,77,78,79,80,81,82,83,84,85,86,87].

### 2.5. Establishment of Novel Food Status

For the regulatory assessment aimed at determining the novel food status of BSG and BRs, the following sources were consulted and evaluated:

- European Commission. Website: Consultation process on novel food status [88];
- European Commission. Website: EU Novel Food Status Catalogue [89];
- Commission Implementing Regulation (EU) 2017/2470 of 20 December 2017 establishing the Union list of novel foods in accordance with Regulation (EU) 2015/2283 of the European Parliament and of the Council on novel foods (Commission Implementing Regulation (EU) 2017/2470 of 20 December 2017 establishing the Union list of novel foods in accordance with Regulation (EU) 2015/2283 of the European Parliament and of the Council on novel foods [90];
- Federal Food Safety and Veterinary Office (FSVO). Website: Authorization of Novel Foods [91].

### 2.6. Statistical Testing

The statistical analysis was performed using RStudio (Version 4.2.1). First, data were tested for normality with the Shapiro–Wilk test. If normality was confirmed, homogeneity of variances was assessed with Bartlett’s test. When both tests returned *p*-values > 0.05, an ANOVA was conducted to detect significant differences among extrudates. Where significant effects were found, pairwise t-tests were applied as post hoc comparisons, using the Bonferroni correction. In cases of non-normality and/or variance heterogeneity, the Kruskal–Wallis test was used, followed, if significant, by Dunn’s post hoc test. Finally, data were visualised in RStudio using subplots, with significance groups indicated by letters.

## 3. Results and Discussion

### 3.1. Characterisation of Raw Materials

Table 2 summarises the nutritional profile and food safety indicators of the side streams.

BSG showed a higher fat content than BRs (7.36% vs. 0.62%). The literature values for BSG range from 5.54% [92] to 10.6% [93]. Studies on germinated barley foodstuff report higher fat contents of 10.2% [93] and 9.2% [94]. However, germinated barley foodstuff can compositionally differ from isolated rootlets, limiting direct comparison with BRs. For BRs, Hegazi et al. reported 1.8% [95], broadly in line with our measurements. The manufacturer of the PPI reports a fat content of 9.1%, which is higher than the side streams and above literature values of 0.5–5.5% [96,97,98,99].

Protein concentrations of BSG and BRs are comparable (21.9% and 24.3%). Literature values for BSG range from 24.0% [93] to 31.07% [92], comparable but slightly higher than that measured in this study. For BRs, Hegazi et al. found 25.0% [95] protein, which is in line with the present results. For PPI the specifications states a protein content of 77.9%, almost within literature ranges of 81% to 95% [96,98,99,100,101,102].

For BSG, the insoluble dietary fibre content is markedly higher (52.4%) than for BRs (33.2%). However, both side streams predominantly contain insoluble fibre. The analysed results are in line with the literature for BSG, where values ranging from 59.0% [93] to 60.56% [92] were found. For BRs, similar values were found for germinated barley foodstuff (34.0% [93]–35.0% [94]). In contrast, the present PPI contains only 2.97% dietary fibre.

The NDMA result for BRs (<0.8 µg/kg) is below the few positive findings reported for un-malted cereal products (2.6–4.2 µg/kg [103]) and far below historical hotspots observed in some malts (10–86 µg/kg [46]) prior to industry mitigations. It is also lower than the ppb levels typically present in finished beers [46]. This confirms that NDMA is not of concern in the present BRs.

Mycotoxins were below detection limits in the present samples, although brewing by-products are often reported to contain them [104].

BSG showed no concerning microbial activity, whereas BRs showed high amounts of aerobic mesophilic bacteria, yeasts and mould. However, these values are mitigatable through processing and should be assessed in the final product.

Table 3 displays the results of the technofunctional analyses, as well as the colour analyses of the three main raw materials.

BRs showed the lowest moisture content of the three raw materials at 5.01 ± 0.11%. BSG and PPI had slightly higher moisture contents at 7.16 ± 0.11% and 7.33 ± 0.14%, respectively. For BR, Neylon et al. reported moisture values of 8.2–12.9% after kilning [3]. As BRs are hygroscopic [105], they are prone to fluctuations in moisture content. Naibaho & Korzeniowska examined eight BSG samples from various breweries, dried by convective drying at 70 °C, and reported moisture contents of 2.2 to 4.4% [13]. The higher value measured in this study may reflect differences in drying methodology.

The WHC analysis showed that PPI had the highest WHC among the three raw materials at 366.6% (±7.2%), closely followed by BSG at 345.2% (±7.9%) and BRs at 276.0% (±1.7%). These values lie within ranges reported in the literature. The BSG samples analysed by Naibaho & Korzeniowska resulted in WHC values between 305.5% and 434.6% [13]. For PPI, the literature likewise reports comparable values: Shen et al. reported 266% [106] and Schumacher et al. a range from 252% to 519% [107], whereas Ma et al. reported as high as 514% [108]. For BRs, no comparative data were found. Differences in WHC among the raw materials can be attributed to factors such as macronutrient composition, processing, and particle size. For PPI, the extraction method in particular can have a major impact on WHC [97]. Stone et al. reported WHC values for PPI from 30% to 360% [97], with differences solely due to different extraction methods. Therefore, extraction-related variability is a likely driver of the WHC differences observed here and must be considered when interpreting these results. BSG and BRs both have relatively high dietary fibre contents (55.0% and 38.7%, respectively), which likely contributes to their high water-holding capacity, as dietary fibre structure and composition are key determinants of hydration properties such as WHC [109]. In contrast to WHC, OHC was less pronounced in the present materials. BSG showed the highest OHC of 178.5% (±18.6%), followed by BRs with 135.9% (±4.0%) and PPI 107.6% (±11.1%). Studies in the literature report OHC ranges of 191.6–221.9% [13] for BSG and 100–200% [97,107] for PPI, aligning with the higher OHC measured for BSG.

WA was lowest for BSG (191.5% ± 14.3%) and highest for BRs (413.5% ± 12.6%), while PPI was in the middle (311.7% ± 7.5%). No comparative data were found in the literature.

The pH values measured differed markedly across materials: PPI 7.01 (neutral), BSG 4.41 (acidic), and BRs 6.07 (slightly acidic). Product specifications did not disclose extraction method or any pH adjustment, although processing steps are known to influence ingredient pH. The neutral pH of PPI aligns with commercial isolates, as Schumacher et al. reported native pH values between 5.50 and 8.15 [107], and may depend on the extraction and neutralisation conditions used. For BSG, Ayissi et al. reported a mildly acidic pH of 5.6 for BSG [110], and Castro and Colpini observed a pH of 5.5 [111], which corresponds with the optimal pH of 5.1–5.6 in beer brewing [112]. The BSG showed greater acidity than reported in the literature, potentially due to residual organic acids, a delayed fermentation of the material before it was dried or the use of preservative stabilisers. BR pH is highly sensitive to kilning conditions and embryo activity during malting, ranging from about pH 5.83 in raw barley to around pH 5.66 in finished malt [113], broadly consistent with the present findings.

Clear differences in PSD were observed between PPI and the side streams. The side streams are similar in X_10_ but differ markedly in X_50_: BRs show nearly double the median (X_50_) of BSG, but the difference narrows at X_90_, with BSG (238.8 ± 10.5 µm) remaining slightly smaller than BRs (274.2 ± 4.2 µm). For PPI, X_10_ is not as small as in the side streams; however, the overall PSD spread is much narrower, as its X_90_ is less than half that of the side streams. This yields a more homogeneous and uniform PSD compared with the two side stream materials. Particle size is known to influence perceived colour, most evident in the lightness (L*). Reducing particle size generally produces a finer, more homogeneous surface texture and increases specific surface area, which enhances light reflection while reducing absorption, yielding a visibly lighter appearance [114]. This phenomenon has been repeatedly reported across foods: decreasing particle size increased L* in mango pulp powder [115], showed a comparable effect in extruded BSG [115] and correlated with increased brightness in bread as mean flour particle size decreased [116].

Correlating with the probable impact of particle size, L* is highest for PPI, which also has the smallest particle size. Nevertheless, for PPI, the measured L* values tend to be lower than those reported by García Arteaga et al. [117] and Jakobson et al. [118], where L* values lie in the range of 85 and 90. However, Ma et al. [108] reported much lower L* values at around 50 for PPI. This discrepancy may be attributable to differences in analysis device, measurement procedures, and pea protein production methods. For BSG, Naibaho & Korzeniowska [13] reported lower lightness (i.e., lower L* values) in the range of 58.436 to 63.732. The BSG a* value of 4.35 ± 0.02 measured in this study lies in the middle of the literature range 3.732–5.326 [13], while the b* value of 14.94 ± 0.05 lies at the lower end of the reported range 14.610–17.116 [13]. Here, too, differences may be due to the use of different instruments, measurement procedures, and BSG processing methods. No comparative literature on BR colour measurements was found.

**Figure 1:**
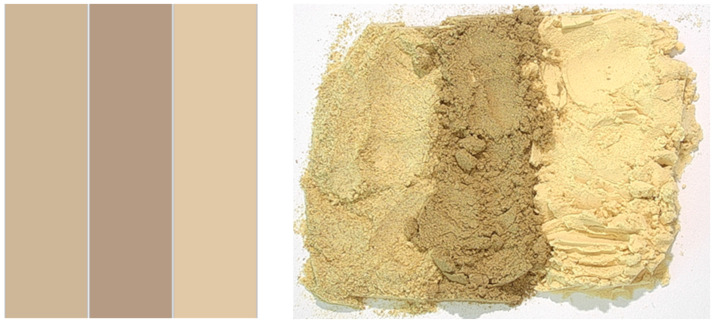
(**Left**): Digital reproduction of colours on basis of the L* a* b* measurements, (**Right**): Macroscopic photographs of raw materials, order: barley rootlets, brewer’s spent grain, pea protein isolate.

Figure 1 shows the digital reproduction of the raw-material hues based on the measured Lab* values and pictures of the raw materials.

### 3.2. Characterisation of HMMA Extrudates

#### 3.2.1. Colour

**Figure 2:**
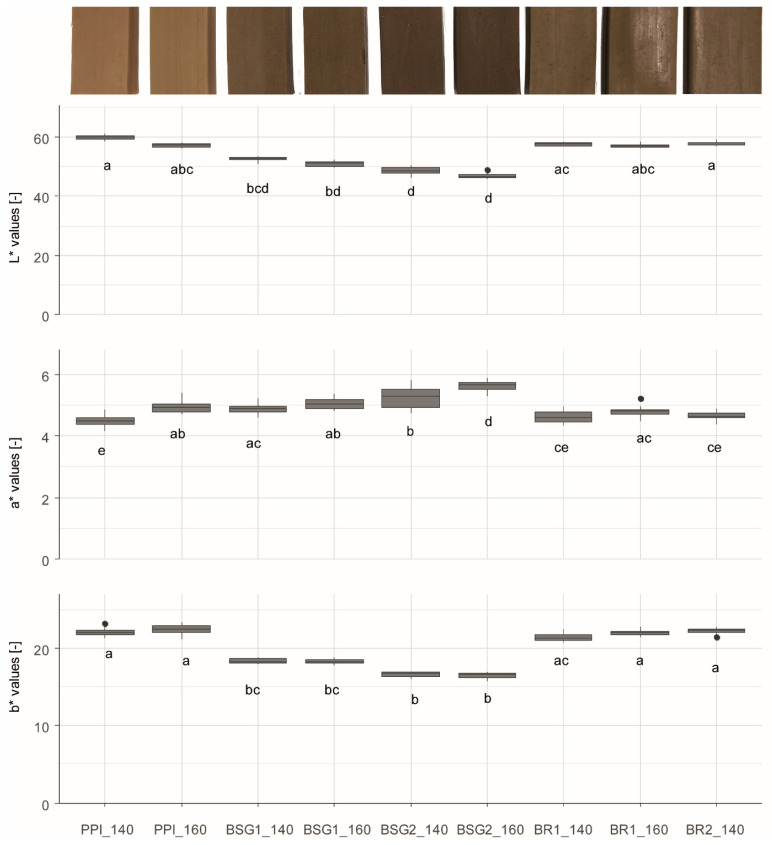
L* a* b* values of extrudates. PPI_140 = 100% pea protein isolate at 140 °C, PPI_160 = 100% pea protein isolate at 160 °C, BSG1_140 = 10% brewer’s spent grain at 140 °C, BSG1_160 = 10% brewer’s spent grain at 160 °C, BSG2_140 = 20% brewer’s spent grain at 140 °C, BSG2_160 = 20% brewer’s spent grain at 160 °C, BR1_140 = 10% barley rootlets at 140 °C, BR1_160 = 10% barley rootlets at 160 °C, BR2_140 = 20% barley rootlets at 140 °C. Compact letters (a, b, c, etc.) indicate groups of significance, i.e., group means not sharing any letter are significantly different with a *p*-value of 0.05.

Figure 2 shows the boxplots of the L* a* b* values plus images of the produced extrudates. ΔE values were calculated and are discussed in the text and displayed in a heatmap in Figure S3 in the Supplementary Material S3, enabling pairwise comparison of all formulations.

Colour analysis of the extrudates shows that all L* values lie between 46.14 and 61.06, all a* values between 4.17 and 5.89, and all b* values between 15.79 and 23.43. PPI_140 showed the highest L* value (59.87 ± 0.72) while BSG2_160 showed the lowest L* value (46.97 ± 0.85). In contrast, a* exhibited the inverse trend: BSG2_160 achieved the highest a* (5.62 ± 0.18), whereas PPI_160 showed the lowest a* (4.48 ± 0.20). Similarly to the L* values, PPI_160 represents the highest median b* value (22.42 ± 0.72), while BSG2_160 holds the lowest b* value (16.51 ± 0.43).

Overall, statistically significant differences were limited and not uniform across temperature. Lightness (L* values) clustered by formulation: the BSG samples showed the lowest L* values, while PPI and BR formulations were among the highest. This correlates with the raw materials’ colour characteristics. Within a formulation, no significant temperature effect emerged. This indicates that formulation, rather than temperature, was the dominant driver of L*. Nevertheless, across formulations there was a consistent, though non-significant, trend toward lower L* at 160 °C compared with 140 °C. In contrast, a* (redness) showed a consistent tendency to be higher at 160 °C than at 140 °C, though this was not significant for all extrudates and no uniform cross-formulation pattern emerged. Within formulations, a significant temperature effect was only observed for PPI and BSG2. Yellowness (b*) was essentially temperature-independent within formulations, with no significant differences visible. Notably, BSG-containing extrudates tended to exhibit lower b* than PPI and some BR formulations, consistent with the lower b* values of the raw material BSG. Overall, formulation effects dominated L*, temperature elevated a* (significantly in selected cases), and b* remained stable across temperatures.

Pairwise comparisons of ΔE values showed that BSG-containing extrudates differed perceptibly from non-BSG samples. Comparing BSG formulations with the other extrudates resulted in ΔE ranges from 5.79 to 14.12. The highest ΔE of 14.12 resulted from the comparison of BSG2_160 and PPI_140. Comparisons between PPI and BR formulations yielded values of 0.44–2.87, indicating slight to noticeable differences.

The observed differences in extrudate colouration can be explained primarily by the colour of the raw materials used and by the process temperature applied during extrusion, with the raw-material colour exerting the greatest influence. The slight differences in colour observed between process temperature and extrudate colour are presumably attributable to the Maillard reaction (non-enzymatic browning) during extrusion. The Maillard reaction can occur over a broad temperature range beginning at approximately 120 °C, with reaction rates increasing markedly between about 140 and 165 °C, which corresponds to temperatures in the melt zone [119,120]. Moreover, the occurrence of the Maillard reaction has been confirmed by several studies, as the formation of Maillard reaction products has been detected during extrusion [121,122]. Sun et al. reported that higher barrel temperature can accelerate the Maillard reaction, leading to more pronounced colouration [38]. Furthermore, the higher fat content of BSG might have additionally enhanced the browning reaction through carbonyl compounds generated via lipid oxidation which then react with amino groups in proteins [123].

#### 3.2.2. Firmness

**Figure 3:**
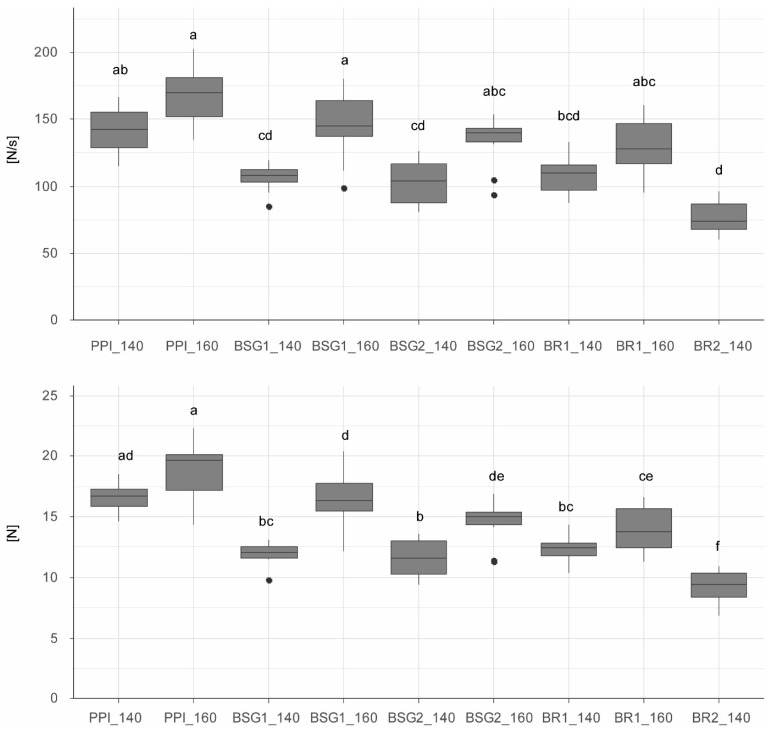
Total work and peak force of extrudates. PPI_140 = 100% pea protein isolate at 140 °C, PPI_160 = 100% pea protein isolate at 160 °C, BSG1_140 = 10% brewer’s spent grain at 140 °C, BSG1_160 = 10% brewer’s spent grain at 160 °C, BSG2_140 = 20% brewer’s spent grain at 140 °C, BSG2_160 = 20% brewer’s spent grain at 160 °C, BR1_140 = 10% barley rootlets at 140 °C, BR1_160 = 10% barley rootlets at 160 °C, BR2_140 = 20% barley rootlets at 140 °C. Compact letters (a, b, c, etc.) indicate groups of significance, i.e., group means not sharing any letter are significantly different with a *p*-value of 0.05.

Figure 3 illustrates maximum force and total cutting work for the extrudates, highlighting statistically significant differences. Maximum force values ranged from 8.39 N to 22.36 N across all samples. These results indicate that both raw materials and operating parameters significantly influence texture. Samples extruded at a maximum process temperature of 160 °C tend to require higher cutting forces than those extruded at 140 °C. A significant difference in maximum force between temperatures within the same dry-matter formulation was observed for recipes with BSG; however, not for those containing BRs. Increasing the BSG substitution level from 10% to 20% did not significantly affect maximum force at a given temperature. By contrast, substituting 20% BRs at 140 °C led to a significantly lower mean firmness of 9.31 N compared with the 10% BRs sample (12.31 N). For the 100% PPI reference extrudates, mean (±SD) maximum forces were 16.62 ± 1.16 N at 140 °C and 18.71 ± 2.44 N at 160 °C. These values are comparable to those in the literature, as Barnés-Calle et al. reported values of 12.40 N at 138 °C and 15.57 N at 159 °C [39].

Observed differences within a formulation may be attributed to non-identical process parameters, such as temperature and water feed. Extrudates exhibited different moisture contents, which is discussed below. The increased hardness observable at 160 °C is likely driven by the lower end-product moisture, consistent with reports that HMMA texture and cutting resistance decrease as moisture increases [20]. Lower screw speed, at equal moisture, can increase hardness [44]; however, screw speed was held constant in this study.

Differences in firmness among extrudates produced at the same maximum temperature can be explained by raw-material composition and by resulting small variations in water feeds required during processing. Relative to PPI, BSG and BRs contain less protein and more dietary fibre and carbohydrates. While PPI contains 77.9% protein (according to the manufacturer), BSG contains only 21.9% and BRs 24.3%. Significant differences in firmness were observed between extrudates made entirely from PPI and those with any side stream addition at 160 °C. At 140 °C, 100% PPI extrudates also differed significantly in maximum force from the substituted extrudates produced under the same temperature conditions. As side stream addition increases, the protein content of the dry mass decreases, affecting the microstructure (i.e., fibrousness), which may explain the lower maximum force. The association of lower protein content with reduced maximum force has likewise been reported by Plattner et al. and Zhang & Ryu: decreasing protein content reduced product firmness and toughness, leading to lower cutting resistance [124,125]. The underlying mechanism is that less protein is available to form intermolecular interactions, leading to reduced aggregation and alignment during extrusion, which results in a reduced ability to form dense fibrous networks responsible for firmness.

#### 3.2.3. Macroscopic Analysis

**Figure 4:**
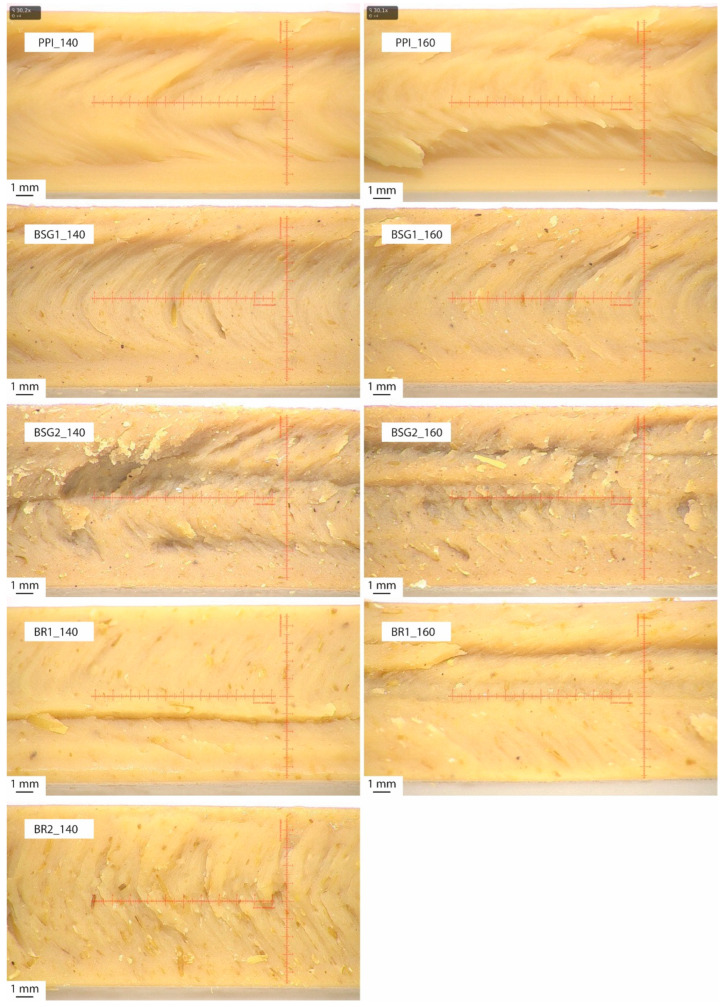
Macroscopic images of the fresh extrudates. PPI_140 = 100% pea protein isolate at 140 °C, PPI_160 = 100% pea protein isolate at 160 °C, BSG1_140 = 10% brewer’s spent grain at 140 °C, BSG1_160 = 10% brewer’s spent grain at 160 °C, BSG2_140 = 20% brewer’s spent grain at 140 °C, BSG2_160 = 20% brewer’s spent grain at 160 °C, BR1_140 = 10% barley rootlets at 140 °C, BR1_160 = 10% barley rootlets at 160 °C, BR2_140 = 20% barley rootlets at 140 °C.

The optical comparison of the extrudates’ fibre structure, photographically captured in Figure 4, reveals clear differences in fibre length, fibre orientation, fibre thickness and the visibility of the fibrous network. PPI_160 displays stronger fibre formation than PPI_140, with thicker and more pronounced fibres, indicating that process temperature affected fibre formation. This observation may be attributed to a higher fraction of denatured proteins at elevated temperatures, which accelerates the disruption of native protein structures. Such structural disruption, including the rearrangement of disulphide bonds, increases molecular mobility and enables protein chains to align in the direction of flow, thereby promoting molecular reorganisation and favouring the development of fibrous structures [126].

In addition, higher processing temperatures increase the melt temperature within the extruder, which reduces melt viscosity and may intensify velocity gradients in the cooling die. This alteration in flow conditions contributes substantially to the development of the fibre structure by enhancing phase separation prior to structure fixation during cooling [27,28].

The extrudates containing side streams did not confirm these observations. In contrast, BSG1_140 displayed thicker, more pronounced fibres than the corresponding extrudate produced at 160 °C (BSG1_160). With an increasing inclusion of BSG (BSG2_140 and BSG2_160) far fewer fibres were visible; where present, they appeared finer and less consistent. Furthermore, the flow marks from the breaker plate were clearly visible in these samples. For the extrudates containing BRs, at both 140 and 160 °C, the fibre structures were less distinct than in the BSG-containing samples. The observable fibres were very fine and layered. Parts of these extrudates exhibited a spongy appearance, indicating less pronounced fibre formation.

Compared with the PPI reference extrudates, the weaker fibre development is likely to be due to the lower protein content resulting from the use of side streams with less protein. Studies found that higher protein content tends to enhance the formation of chemical cross-links, which in turn promotes fibrous structure formation [30,125]. Moreover, the higher protein content of PPI may intensify the thermo-gelation effect, further contributing to a meat-like texture [30]. Substitution with BSG and BRs also introduces a second biopolymer phase with higher dietary fibre content. Van Der Sman & Van Der Goot [28] examined this phenomenon using blends of soy protein isolate and soy protein concentrate and found an interaction between the presence of a second biopolymer phase and syneresis, which can alter the thickness of the V-shaped lamellae.

The spongy consistency of the BR extrudates may be attributable to the low WHC of BRs. Zhang et al. reported a comparable spongy structure in plant-protein-based HMMA extrudates when the moisture content was high [41]. Although the PPI extrudates and the BR-substituted variants had very similar product moistures (57.13% to 60.31%), the lower WHC of BRs could lead to more free water in the matrix. This may promote the spongy consistency, analogous to the effects of excessive moisture [41].

An internally conducted preliminary sensory screening of the extrudates using an expert assessment with four panellists according to DIN 10975 broadly confirmed the instrumental findings on structure and texture. Extrudates processed at 140 °C were generally perceived as more fibrous with longer fibres. Higher substitution levels, especially 20%, were associated with reduced elasticity, more pronounced disintegration of the fibre structure and a weaker, crumblier matrix. Overall, process temperature had a smaller impact on perceived juiciness than the type of side stream used: extrudates containing BSG were rated as comparatively dry and crumbly, while BR-containing extrudates were additionally characterised by a distinct fishy off-flavour.

#### 3.2.4. Final Composition

In Table 4, the analysed moisture contents of the extrudates and the calculated nutritional values are displayed. Moisture analysis showed a consistent trend: extrudates processed at the lower temperature (140 °C) exhibited higher moisture contents. For the higher-temperature conditions, the water feed was consistently lower due to operational constraints, which explains the observed differences in the final product. The nutritional values were calculated considering the water loss during processing, i.e., based on measured moisture content of the final products. Substituting PPI with BSG or BRs resulted in an increase in fibre content and decrease in protein content. This observation is in accordance with the raw-material composition. Due to the fibre addition of the side streams, all extrudates containing BSG or BRs can be labelled “source of fibre”; moreover, the variants BSG2_140 and BSG2_160 can be labelled “high-fibre” [126]. Furthermore, according to Swiss and EU legislation [127,128], even taking into account a minimal change in the product composition during pan frying, the extrudates meet the criteria for a "high-protein" claim.

Linking the final composition to the observed product quality, it can be deduced that a lower protein content leads to less cross-linking and reduced development of a fibrous structure, both of which lead to a reduction in the firmness and elasticity of the corresponding end products. Furthermore, the increasing fibre content at higher inclusion levels of side streams BRs and BSP lead to a disrupted protein network, which results in a more brittle and crumbly structure.

### 3.3. Environmental Assessment

**Figure 5:**
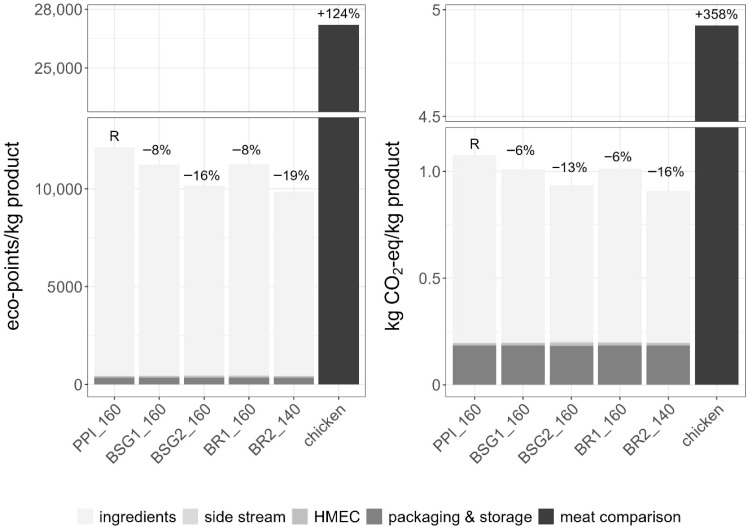
Overall environmental impacts (eco-points, **left**) and life cycle greenhouse gas emissions (kg CO_2_-eq, **right**) of 1 kg product with partial substitution of pea protein by BRs and BSG, assessed using the Ecological Scarcity Method and the IPCC 2021 methodology. Ingredients: production of pea protein and other ingredients, side stream: BRs and BSG economic allocation and pre-processing including drying and milling for the scenarios, HMEC: high moisture extrusion cooling, packaging and storage: material and cooled storage. PPI_160 (R): 100% pea protein isolate at 160 °C, BSG1_160: 10% brewer’s spent grain at 160 °C, BSG2_160: 20% brewer’s spent grain at 160 °C, BR1_160: 10% barley rootlets at 160 °C, BR2_140: 20% barley rootlets at 140 °C.

To determine whether BRs and BSG can outperform pea protein environmentally, their substitution potential was assessed (Figure 5). The reference product used for this study is PPI_160, consisting of 100% pea protein, which results in climate impacts of 1.08 kg CO_2_-eq per kg product. Across the four extrudate formulations with BRs and BSG, the climate impacts range from 0.91 to 1.01 kg CO_2_-eq per kg of extrudate (Figure 5 right and Table S16 in Supplementary Material S2). When comparing the results to existing LCAs, it lies within the range of 0.5–2.4 kg CO_2_-eq per kg extrudate product [129]. The dominant contributor in this study is the ingredient stage, accounting for 78.3% to 81.8% of total impacts. In particular, pea protein production, driven by pea production and energy-intensive wet fractionation, exhibits the highest burden. In contrast, the side streams (BSG and BRs), including their pre-processing steps (drying and milling) and the economic allocation, contribute <1% in all recipes. Their economic value is very small compared to beer, and therefore only a very small share of the environmental burden from beer production is allocated to BSG and BRs. However, economic allocation is inherently sensitive to market dynamics. Changes in demand or increased valorisation of side streams can lead to higher prices and thus alter allocation factors. Consequently, the results reflect current market conditions and should be interpreted with caution, as the environmental benefits may vary under different economic scenarios.

Substituting pea protein with BRs or BSG therefore results in meaningful climate impact reductions: 6% reduction at a 10% substitution level and 13–16% at 20%. This pattern reflects a general trend in LCAs of alternative proteins, where reducing dependence on purified protein isolates substantially decreases environmental impacts, especially when these isolates are replaced with low-burden side streams [130]. The Ecological Scarcity assessment confirms the same trend (Figure 5 left and Table S17 in Supplementary Material S2).

The high-moisture extrusion cooking (HMEC) stage contributes 0.012 kg CO_2_-eq per kg extrudate, corresponding to ~1.3% of the total impacts of the four side stream recipes. This is substantially lower than the ~20% extrusion contribution reported in the study on plant-based meats produced by high-moisture extrusion [59]. The discrepancy is primarily driven by the electricity mix. Replacing the Swiss electricity mix, used in our assessment, with a European mix (ENTSO-E) increases the HMEC contribution from ~1% to ~15% (see Figure S2 in Supplementary Material S2). The reduction potential therefore decreases by 14%. This highlights the strong sensitivity of extrusion impacts to the electricity mix. Consequently, the environmental benefits identified in this study are context-specific and most pronounced in regions with low-carbon electricity systems. In regions with more carbon-intensive electricity mixes, the mitigation potential is significantly reduced. This limits the direct transferability of results across geographical contexts and underlines the importance of using region-specific energy data in LCA studies of novel food processing technologies.

As an additional comparison, chicken meat is included as a conventional benchmark. The plant-based extrudates evaluated here show climate impacts roughly 4.5 times lower than chicken, underscoring their environmental advantage beyond the internal substitution of pea protein.

Overall, using BSG or BRs to replace pea protein reduces climate impacts by 6–16% and total environmental impacts by 8–19% compared to the pea protein-based reference product (PPI_160). Higher substitution rates increase this reduction potential. In addition, the use of low-carbon electricity is essential for minimising impacts from energy-intensive processes such as HMEC.

**Figure 6:**
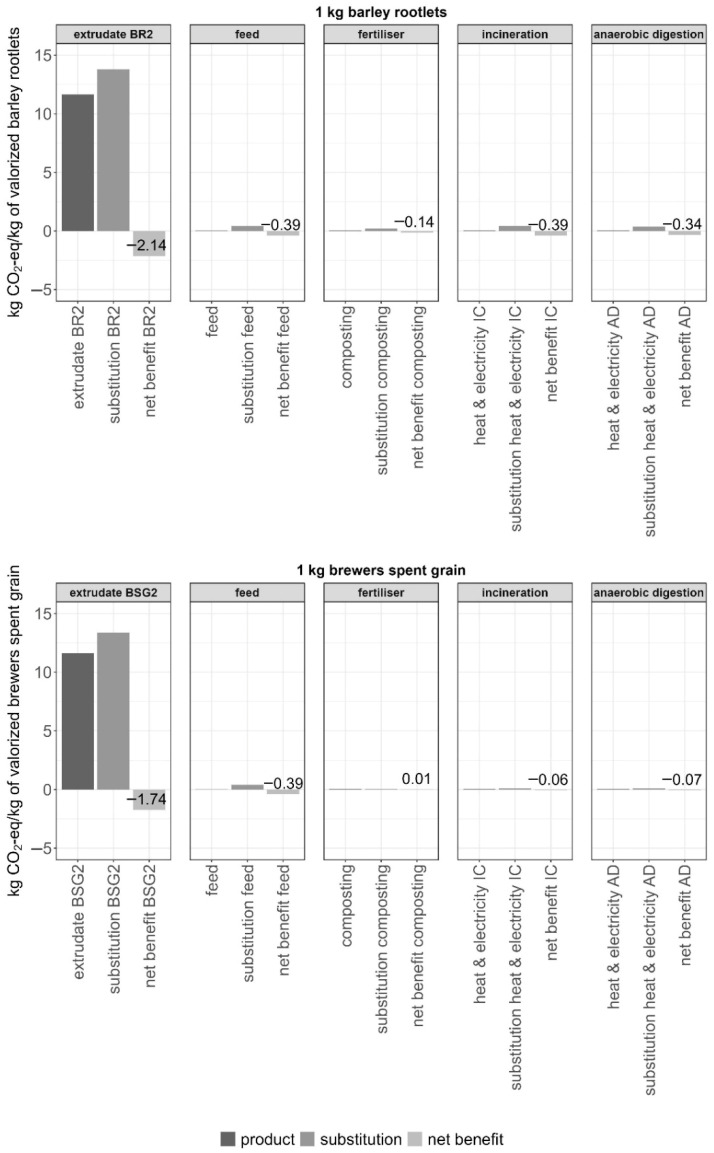
Life cycle greenhouse gas emissions (kg CO_2_-eq, right) of barley rootlets (BRs) and brewer’s spent grain (BSG) valorisation and utilisation pathways. The assessment was carried out using the IPCC 2021 methodology applying a system expansion approach. The results include the climate impact in CO_2_-eq per kilogram of product that incorporates valorised side streams (product), per kilogram of substituted product (substitution) and the net benefit between product and substitution (net benefit). Substitution assumptions: mineral fertiliser with equivalent N–P–K content for composting; Swiss average electricity mix and natural gas (central/small-scale) for incineration and anaerobic digestion; wheat bran, proti-grain DDGS (Distillers Dried Grains with Solubles) and sunflower cake as nutrient-equivalent feed; recipe PPI_160 for BSG2 and BR2. BSG2: 20% Brewer’s spent grain at 160 °C. BR2: 20% Barley rootlets at 140 °C.

To evaluate whether the food valorisation of brewery side streams yields the greatest environmental benefits, multiple utilisation scenarios were compared. In addition to extrudate production, both BRs and BSG can be valorised as animal feed, compost, or through energy recovery via incineration or anaerobic digestion (Figure 6). A system expansion approach was applied to quantify environmental impacts, substitution effects, and the resulting net climate benefits per kilogram of BRs and BSG.

Among all pathways, incorporation into extrudates provided the largest climate gains, with a net saving of 2.14 kg CO_2_-eq per kg of used BRs and 1.74 kg CO_2_-eq per kg of used BSG (see Tables S18 and S19 in Supplementary Material S2). In contrast, alternative applications delivered only marginal benefits. Incineration, based on a lower heating value of 7.1 MJ/kg wet BSG and 17.94 MJ/kg BR, generated 0.06 and 0.39 kg CO_2_-eq respectively. Using BRs and BSG as animal feed can result in a net saving of 0.39 kg CO_2_-eq. However, when BSG is used in extrudates and therefore no longer available as animal feed, conventional feed must be produced instead, which reduces the climate benefit. Under this assumption, the climate benefit decreases by 23.6%, from 1.74 to 1.33 kg CO_2_-eq per kg of BSG used.

In conclusion, the results show that valorising BSG and BRs in human food products delivers the largest environmental benefits, with reductions of up to 16% in climate impact and up to 19% in overall environmental impact, particularly at higher inclusion rates and under low-carbon electricity conditions. This reinforces the potential of brewery side stream valorisation as a meaningful strategy for reducing the environmental footprint of plant-based foods.

### 3.4. Novel Food Status

According to the EU Novel Food Status Catalogue [89], BSG is not novel, as this product was consumed to a significant degree within the Union before 15 May 1997 and no production process not used for food production within the Union before 15 May 1997, which gives rise to significant changes in the composition or structure of the food, affecting its nutritional value, metabolism or level of undesirable substances, has been applied.

The Novel Food Status Catalogue further states that BSG predominantly consists of fibre-rich and proteinaceous fractions derived from the residual malt remaining after the beer brewing process, and that it contains only minimal amounts of chaff. The chaff, when used as an isolated ingredient, is considered as novel. In addition, it can be noted that BSG fibre and BSG protein, two fractions gained from BSG, have a separate entry in the EU Novel Food Status Catalogue; they are also classified as not novel [88,89].

BRs are classified as not novel in food supplements according to the EU Novel Food Status Catalogue [89] as the respective entry in the Catalogue under “*Hordeum vulgare* L.” includes young branches, roots and fruits. However, uses in food categories other than food supplements may be considered novel and may therefore require authorisation under the legal framework for novel foods. According to Commission Implementing Regulation (EU) 2017/2470 (“Union list of novel foods” [90]), no authorisation has been granted yet for BRs to be used in foodstuffs other than food supplements.

In Switzerland, neither BSG nor BRs have been classified as novel or not by the competent authority, the Federal Food Safety and Veterinary Office (FSVO), as no applications have been submitted. However, Switzerland acknowledges the novel food status classifications established in the EU [91].

## 4. Conclusions

Partial substitution of PPI with BSG or BRs in HMMAs is feasible but entails clear quality trade-offs. The inclusion of BSG significantly darkened the extrudates, while the use of BRs resulted in similar L* values as the PPI extrudates. Redness (a*) tended to increase at 160 °C, whereas yellowness (b*) did not change systematically. Firmness rose when end-product moisture was lower and decreased with the inclusion of either BSG or BRs, possibly attributed to less fibre formation with lower protein content. The macroscopic analyses of the fibre morphology further confirmed the less pronounced structurisation of the HMMAs containing BSG or BRs. Overall, valorisation of BSG and BRs in HMMAs is feasible. However, their effects must be carefully assessed to ensure that target sensory properties are achieved. Due to their distinct fishy taste and fine fibre structure, BR-containing samples may be a suitable basis for a tuna substitute, while the BSG-based samples are more neutral in taste. Given the achieved reductions of up to 16% in climate impact and 19% in overall environmental impact, the integration of BSG and BRs into HMMAs is a viable valorisation strategy. Future work should focus on optimising moisture content and sensory properties. While BSG is not classified as novel food, uses of BRs for purposes other than in or as food supplements may be considered novel and the BR-containing extrudate would therefore require authorisation under the legal framework for novel foods before the product could legally be placed on the market.

## Figures and Tables

**Table 1:** Extrudate sample composition of pea protein isolate (PPI), brewer’s spent grain (BSG) and barley rootlets (BRs) inclusive water feed settings and maximum barrel temperature (Max. Barrel Temp.) applied during processing.

Sample Code	PPI [%]	BSG [%]	BR [%]	Water [g/min]	Max. Barrel Temp. [°C]
PPI_140	100	0	0	29.9	140
PPI_160	100	0	0	27.9	160
BSG1_140	90	10	0	29.9	140
BSG1_160	90	10	0	27.1	160
BSG2_140	80	20	0	29.3	140
BSG2_160	80	20	0	26.5	160
BR1_140	90	0	10	27.9	140
BR1_160	90	0	10	27.1	160
BR2_140	80	0	20	27.9	140

**Table 2:** Nutritional and safety characterisation of brewer’s spent grain and barley rootlets (nd: not detected, na: not analysed).

	Brewer’s Spent Grain		Barley Rootlets	
	Mean	SD	Mean	SD
Fat [%]	7.36	0.16	0.62	0.02
Protein [%]	21.9	1.1	24.3	1.3
Dietary fibres (total) [%]	55.0	7.1	38.7	5.0
Dietary fibres (soluble) [%]	2.6	0.3	5.4	0.7
Dietary fibres (insoluble) [%]	52.4	8.7	33.2	5.5
NDMA (N-Nitrosodimethylamine) [μg/kg]	na	na	<0.8	na
Total aflatoxins (B1, B2, G1, G2) [μg/kg] *	nd	nd	nd	nd
Ochratoxin A [μg/kg] *	nd	nd	nd	nd
Deoxynivalenol [μg/kg] *	nd	nd	nd	nd
Nivalenol [μg/kg] *	nd	nd	nd	nd
Zearalenone [μg/kg] *	nd	nd	nd	nd
T-2-Toxin [μg/kg] *	nd	nd	nd	nd
HT-2-Toxin [μg/kg] *	nd	nd	nd	nd
Sum Fumonisine [μg/kg] *	nd	nd	nd	nd
Aerobic mesophilic germs [CFU/g]	<1000	na	>3.0 × 10^8^	na
Yeasts [CFU/g]	<100	na	>150,000	na
Mould [CFU/g]	<100	na	9200	na
*Bacillus cereus* [CFU/g]	<100	na	na	na

* Limit of detection for mycotoxins: 0.1 μg/kg for Aflatoxin B1, B2, G1, G2, Ochratoxin A, 1 for Zearalenon, 5 μg for T-2-Toxin and HT-2-Toxin, 10 mg/kg for Fuminisin B1 and B2.

**Table 3:** Functional characterisation of the raw materials brewer’s spent grain (BSG), barley rootlets (BRs) and pea protein isolate (PPI).

	BSG		BR		PPI	
	Mean	SD	Mean	SD	Mean	SD
Moisture content [%]	7.33	0.14	5.01	0.11	7.16	0.11
Water holding capacity [%]	345.2	7.9	276.0	1.7	366.6	7.2
Oil holding capacity [%]	178.5	18.6	135.9	4.0	107.6	11.1
Water absorption [% d.m.]	191.5	14.3	413.5	12.6	311.7	7.5
pH value []	4.41	0.01	6.07	0.01	7.01	0.02
X_10,3_ = 10% particle size [µm]	13.75	0.45	14.02	0.62	25.01	0.41
X_50,3_ = median particle size [µm]	45.47	4.57	92.08	11.93	51.13	0.65
X_90,3_ = 90% particle size [µm]	238.78	10.47	274.25	4.25	106.10	2.20
L* [-]	64.88	0.14	74.43	0.10	81.16	0.14
a* [-]	4.35	0.02	2.89	0.04	2.76	0.02
b* [-]	14.94	0.05	16.94	0.06	18.71	0.08

**Table 4:** Final composition of extrudates. PPI_140 = 100% pea protein isolate at 140 °C, PPI_160 = 100% pea protein isolate at 160 °C, BSG1_140 = 10% brewer’s spent grain at 140 °C, BSG1_160 = 10% brewer’s spent grain at 160 °C, BSG2_140 = 20% brewer’s spent grain at 140 °C, BSG2_160 = 20% brewer’s spent grain at 160 °C, BR1_140 = 10% barley rootlets at 140 °C, BR1_160 = 10% barley rootlets at 160 °C, BR2_140 = 20% barley rootlets at 140 °C.

[g/100 g]	Energy [kcal/100 g]	Fat	Carbohydrates	Fibres	Protein	Salt	Moisture	
							Mean	SD
PPI_140	178.0	4.0	0.1	1.3	34.7	1.0	58.89	1.38
PPI_160	182.4	4.1	0.1	1.4	35.5	1.0	57.89	0.58
BSG1_140	167.4	3.8	0.4	3.5	31.0	0.9	60.31	0.26
BSG1_160	172.5	3.9	0.5	3.6	32.0	0.9	59.10	1.38
BSG2_140	162.2	3.7	0.8	5.7	28.5	0.8	60.49	0.43
BSG2_160	175.0	4.0	0.8	6.2	30.7	0.8	57.37	0.49
BR1_140	174.8	3.7	1.5	2.9	32.5	0.9	58.54	0.24
BR1_160	180.5	3.8	1.5	3.0	33.5	0.9	57.19	0.53
BR2_140	178.1	3.8	1.5	3.0	33.1	0.9	57.77	0.98

## Data Availability

The original contributions presented in this study are included in the article/Supplementary Materials. Further inquiries can be directed to the corresponding author.

## Supplementary Materials

The following supporting information can be downloaded at: https://www.mdpi.com/article/10.3390/foods15081327/s1.
