# Supplementary material for: Upcycling Brewer’s Spent Grain and Barley Rootlets by Partial Substitution of Pea Protein Isolate in Extruded High Moisture Meat Analogues
Source: Foods. 2026 Apr 10;15(8):1327. doi: 10.3390/foods15081327 (PMC13114482; doi:10.3390/foods15081327)
Supplement: Supplementary file 1 [file foods-15-01327-s001.zip › SupplementaryMaterial S2.pdf]

Supplementary Material 02 - Life Cycle Assessment of Brewers' Spent Grain and Barley Rootlets

For the LCA conducted in this study, detailed inventory data were required for each valorization and utilization pathway of brewers' spent grain (BSG) and barley rootlets (BR). Chapter 1 of this supporting material provides the complete life cycle inventory for all scenarios, accompanied by explanatory notes and references to the specific ecoinvent datasets used. Chapter 2 outlines the economic allocation approach, and Chapter 3 presents additional results and visualizations.

BSG and BR are assessed as alternative sources to pea protein in the production of plant-based meat analogues (PBMAs) via high-moisture extrusion cooking (HMEC), yielding high-moisture meat analogues (HMMAs) with anisotropic, fibrous textures. In addition, several other utilization scenarios are modelled: (1) incineration with heat and electricity co-generation, (2) anaerobic digestion for biogas production with energy co-generation, (3) use as animal feed, and (4) composting.

1. Inventory Data

1.1. Valorization Scenario – PBMAs using BSG and BR

The modelling of beer production is based on data from a study of the UK beverage sector [1] as summarized in Table 1. The environmental burdens associated with the side streams BSG and BR are allocated using economic allocation (see Chapter 2).

Table S1: Outputs inputs from and to the technosphere for beer and the side-streams BSG and BR. Datasets are from the ecoinvent v3.11 database.

Outputs to technosphere	Amount	Unit	comment
Beer, at plant {CH} - VALISS	1000	l	
Brewer Spent Grain (BSG) wet - VALISS	94.9	kg	
Barley Rootlets (BR) - VALISS	2.92	kg	

Inputs form technosphere	Amount	Unit	comment
Barley grain, Swiss integrated production {CH}| barley grain production, Swiss integrated production, intensive	73	kg	
Tap water {CH}| market for tap water	8430	kg	
Wheat grain, Swiss integrated production {CH}| wheat grain production, Swiss integrated production, intensive	1.3	kg	
Sodium hydroxide, without water, in 50% solution state {GLO}| market for sodium hydroxide, without water, in 50% solution state	9	kg	
Phosphoric acid, industrial grade, without water, in 85% solution state {RER}| purification of wet-process phosphoric acid to industrial grade, product in 85% solution state	2	kg	
Sulfuric acid {RER}| sulfuric acid production	2.5	kg	
Carbon dioxide, liquid {RER}| market for carbon dioxide, liquid	30	kg	
Heat, district or industrial, other than natural gas {CH}| heat production, light fuel oil, at industrial furnace 1MW	36.6*0.84/ 0.02465 = 1.25E3	MJ	0.02465 kg light fuel oil per MJ oil; density of 0.84 kg/ l

All input data used for modelling the production of plant-based meat analogues (PBMAs) via high-moisture extrusion cooking (HMEC) are summarized in Table 2. Five formulations are presented, each using different proportions of pea protein, BSG, and BR. Both BR and BSG must be processed in dried and milled form; BR is already dry and only requires milling, whereas BSG must be dried prior to milling and further use. The following assumptions are applied to the processing steps:

- Drying: BSG typically contains ~75% moisture. Electricity demand for drying using a conventional electric convective dryer ranges from 1.68–1.80 kWh/kg BSG.[2] The energy required to evaporate 750 g of water per kg BSG via hot-air drying is reported as 1.25 kWh.[3] For modelling purposes, a drying energy demand of 1.7 kWh/kg BSG is adopted.
- Milling: Milling is modelled based on machine operation and electricity consumption.
    - Machine operation based on material properties:
        - Bulk density of BR: 224 kg/m³ (0.224 g/cm³)[4]
        - Bulk density of BSG: 0.15 g/cm³ [5]
    - Electricity consumption:
        - milling is performed using a Retsch SR300, which has a power rating of 2.5 kW (Retsch, 2025). Assuming operation at full load, the energy demand is modelled as 2.5 kWh per milling batch.

- The SR300 has a maximum working volume of 5 L. Using the reported BSG density of 1.33 g/cm^3 for the ground material,[5] the mill can process up to 6.65 kg BSG per batch (1.33 g/cm^3 × 5000 cm^3). The corresponding BR batch mass is derived analogously using its bulk density. All calculations are provided in Table 2.

- High-moisture extrusion cooking (HMEC): The process includes preconditioning with water and heat, extrusion, cooling, and cutting. All stages require energy, which is modelled entirely as electricity based on data from Bonales et al..[6]

Table S2: Inventory data for the valorization scenario of extrudates. Functional unit: extrudates made of 1kg protein input. Datasets are from the ecoinvent database. Calculations for BSG and BR.

	source	dataset & comment	Unit	PPI_160	BSG1_160	BSG2_160	BR1_160	BR2_140
output			*kg*	*2.5603*	*2.5129*	*2.4812*	*2.5129*	*2.5603*
**ingredients**	See Table 1	Brewer Spent Grain (BSG) wet - VALISS	kg	0	0.1	0.2	0	0
	See Table 1	Barley Rootlets (BR) - VALISS	kg	0	0	0	0.1	0.2
	VALISS, Ecoinvent	Water included in recipe<br>Tap water {CH}| market for tap water	kg	1.5453	1.4979	1.4662	1.4979	1.5453
	VALISS, Ecoinvent	beef aroma<br>Curry powder spice {GLO}| market for	kg	0.015	0.015	0.015	0.015	0.015
	See Table 3	pea protein 80%<br>Pea protein powder - 80% protein concentrate (VALISS)	kg	1	0.9	0.8	0.9	0.8
**pre-processing BSG and BR**	Ecoinvent	Transport of BSG and BR to processing site of pea protein<br>Transport, freight, lorry, unspecified {GLO}| market for	tkm	100km *0.001 kg = 0.1tkm	100km *0.001 kg = 0.1tkm	100km *0.001 kg = 0.1tkm	100km *0.001 kg = 0.1tkm	100km *0.001 kg = 0.1tkm
	[2,3] Ecoinvent	Drying energy<br>Electricity, low voltage {CH}| market for electricity, low voltage	kWh	0	0.17	0.34	0	0
	[7] Ecoinvent	Milling machine<br>Industrial machine, heavy, unspecified {RER}| market for industrial machine, heavy, unspecified<br><br>assumptions – milling with sr300 retsch impact mill [7]<br>- machine type: impact mill (sr300, retsch)<br>- assumed lifespan: 15 years<br>- machine weight: 60 kg<br><br>processing BSG:<br>- batch volume: 5 l (5000 cm^3)<br>- density of BSG: 0.15 g/cm^3 [5]<br>- BSG: actual batch mass: 0.75kg<br>- BSG: time per batch: 5 minutes -→ 0.75kg/5min<br>→ processing rate: 9 kg/hour<br><br>annual and lifetime throughput:<br>operating time:<br>- 10 hours/day, 5 days/week, 52 weeks/year → 2,600 hours/year<br>- annual throughput: 9 kg/h×2,600 h=23'400 kg/year<br>- total lifetime throughput (15 years): 23'400 kg/year×15=351'000 kg<br>- value: 60/1'872'000 = 0.000171 kg per kg of BSG	kg	0	0.0000 0321	0.0000 0642	0.0000 114	0.0000 228

		o 0.000171 kg /1000*amount needed<br>o For 100g: 0.0000171 kg<br>o For 200g: 0.0000342 kg<br><br>processing BR:<br>- batch volume: 5 l (5000 cm³)<br>- density of BR: 0.2243g/ cm³ [4]<br>- actual batch mass: 1.12 kg<br>- time per batch: 5 min→ 1.12kg/5min<br>→ processing rate: 13.5 kg/hour<br><br>annual and lifetime throughput:<br>operating time:<br>- 10 hours/day, 5 days/week, 52 weeks/year → 2,600 hours/year<br>- annual throughput: 13.5 kg/h×2,600 h=35’100 kg/year<br>- total lifetime throughput (15 years): 35’100 kg/year×15=526’500 kg<br>- value: 60/526’500 = 0.000114 kg per kg of BR<br>o 0.000114 kg /1000*amount needed<br>o For 100g: 0.0000114 kg<br>o For 200g: 0.0000228 kg						
	7	Milling energy<br>Electricity, low voltage {CH}| market for electricity, low voltage<br><br>milling with sr300 retsch impact mill [7]<br>- Max: 2.5kW<br>- 2.5 kWh for<br>o BSG: 9kg/h → 2.5/9 = 0.28 kWh for 1kg BSG<br>o BR: 13.5kg/h → 2.5/13.5 = 0.19 kWh for 1kg BR	kWh	0	0.28/10 = 0.028	0.28/10*2 = 0.056	0.19/10 =0.019	0.19/10*2 = 0.038
**high moisture extrusion cooling**	6	Pre-conditioning energy<br>Electricity, medium voltage {CH}| market for electricity, medium voltage	kWh	0.0263	0.0263	0.0263	0.0263	0.0263
		Pre-conditioning water<br>Tap water {CH}| market for tap water	g	2867	2867	2867	2867	2867
		Heat from natural gas<br>Heat, central or small-scale, natural gas {CH}| market for heat, central or small-scale, natural gas	MJ	0.11	0.11	0.11	0.11	0.11
		Water heating energy<br>Electricity, medium voltage {CH}| market for electricity, medium voltage	kWh	0.0316	0.0316	0.0316	0.0316	0.0316
		Extrusion energy<br>Electricity, medium voltage {CH}| market for electricity, medium voltage	kWh	0.8153	0.8153	0.8153	0.8153	0.8153
		Cooling energy<br>Electricity, medium voltage {CH}| market for electricity, medium voltage	kWh	0.0053	0.0053	0.0053	0.0053	0.0053
		Cutting energy<br>Electricity, medium voltage {CH}| market for electricity, medium voltage	kWh	0.5786	0.5786	0.5786	0.5786	0.5786
		Machinery usage<br>Industrial machine, heavy, unspecified {RER}| market for industrial machine, heavy, unspecified<br>Assumptions: [8]<br>- machine type: High-Moisture Meat Analogues extruder<br>- assumed lifespan: 15 years<br>- machine weight: 1500 kg<br>- troughput: 500 kg/h<br><br>Calculations:<br>- runs 10H/day, 5 days a week, 52 weeks/year, 15 years → 500*10*5*52*15 = 19’500’000 kg/lifetime	kg	*2.5603 *7.7e-5 = 0.000197*	*2.5129 *7.7e-5 = 0.0001933*	*2.4812 *7.7e-5 = 0.000191*	*2512.9 *7.7e-5 = 0.0001933*	*2.5603 *7.7e-5 = 0.000197*

		- 1500 kg weight/19'500'000 kg product = 7.7e-5/kg product						
**packaging & sale**	[9]	Plastic<br>Packaging film, low density polyethylene {GLO}| market for<br>- According to UN, plastic packaging (PE) is the most used packaging for meat alternatives<br>- 30 g plastic per kg meat alternative<br>- 2.5129 kg *0.03kg (calculated for each output amount)	kg	0.0768	0.0754	0.0744	0.0754	0.0768
		Forming<br>Injection moulding {GLO}| market for<br>Same values as for the PE amount	kg	0.0768	0.0754	0.0744	0.0754	0.0768
		Storage at 6°C<br>Operation, reefer, cooling {GLO}| operation, reefer, cooling, 40-foot, high-cube, carbon dioxide, liquid as refrigerant<br>Assumption: 5 days	kg* day	*2.5603 *5 = 12.8*	*2.5129 *5*	*2.4812 *5*	*2.5129 *5*	*2.5603 *5 = 12.8*
**Waste**		Wastewater<br>Wastewater, average {CH}| market for wastewater, average	m³	0.0028<br>67	0.0028<br>67	0.0028<br>67	0.0028<br>67	0.0028<br>67

As the substitution or reference product for the extrudates, the purely pea-based extrusion meat analogue (PPI_160) is used. Input data for pea protein are provided in Table 3. For contextual comparison, real chicken meat is also included. The chicken comparison is based on the following datasets: *chicken, meat, unallocated, at slaughterhouse/GLO U based on the ecoinvent dataset Chicken for slaughtering, live weight {GLO}| market for chicken for slaughtering, live weight.*[10]

Table S3: Input data for modelling pea protein powder with 80% protein concentration. Datasets are from the ecoinvent database.

**Outputs to technosphere**	**Amount**	**Unit**	**comment**
Pea protein powder - 80% protein concentrate (VALISS)	1	kg	

**Inputs form technosphere**	**Amount**	**Unit**	**comment**
Peas<br>*Protein pea {GLO}| market for protein pea based on → Protein pea {FR}| protein pea production*<br><br>Protein pea, Swiss integrated production {CH}| protein pea production, Swiss integrated production, intensive	4	Kg	Moisture content of 13% at storage → already dried<br>[11–13]
Energy for protein concentrating<br>Electricity, low voltage {CH}| market for electricity, low voltage	0.187	MJ	[13]
Heat energy of Pea dry crushing<br>Heat, central or small-scale, natural gas {CH}| market for heat, central or small-scale, natural gas	2.5	MJ	[11,12]<br><br>460 MJ for 709kg pea meal. 1kg pea concentrate needs 4 kg pea meal -> 460/709*4
Electricity of Pea dry crushing<br>Electricity, low voltage {CH}| market for electricity, low voltage	1.18	kWh	[11,12]<br><br>209 kWh for 709kg pea meal. 1kg pea concentrate needs 4 kg pea meal -> 460/709*4
Transport	0.4	tkm	4kg for 100km

Transport, freight, lorry, unspecified {GLO}| market for			

Outputs to technosphere: waste treatment	Amount	Unit	comment
Biowaste {CH}| treatment of biowaste, industrial composting	3	kg	
Wastewater of Pea dry crushing<br><br>Wastewater, average {CH}| market for wastewater, average	0.58	l	[11,12]<br><br>104 l for 709kg pea meal. 1kg pea concentrate needs 4 kg pea meal -> 104/709*4

1.2 Utilization Scenario 1 – incineration of BSG and BR

The scenario incineration is based on the approach of Beretta et al. (2017). For the life cycle inventory of the incineration, an existing eco-inventory from the Ecoinvent database for the incineration of biowaste was used for all by-streams: *Biowaste {GLO}| treatment of biowaste, municipal incineration.* During incineration, heat and electricity are generated. The amount of produced electricity and heat was determined proportionally to the lower heating value.

The heating value was calculated considering the nutrient composition using following equation:

$$H_u = P * B_P + F * B_F + C * B_C + Fi * B_{Fi} - W * B_W$$

With P = protein content, F = fat content, C = carbohydrate content, F_i = fiber content, W = water content, B_i = physical calorific value of the nutrient

Table S4: physical calorific value per nutrient and the content per kg BSG and BR of these nutrients. Calculations for BSG and BR.

Nutrition	physical calorific value of the nutrient [MJ/kg]	nutrition per kg		
		**BSG dried**	**BSG wet**	**BR**
P = protein content	23 MJ/kg	0.219	0.0641	0.243
F = fat content	38.9 MJ/kg	0.074	0.0217	0.006
C = carbohydrate content	17.2 MJ/kg	0.011	0.0032	0.289
F_i = fiber content	18 MJ/kg	0.550	0.161	0.387
W = water content	-2.441 MJ/kg	0.048	0.750	0.075

Based on the values in Table 4, the equation can be calculated for BSG (dried and wet) and BR:

$$H_{u,dried} = 0.219kg * 23\frac{\mathrm{MJ}}{\mathrm{kg}} + 0.074kg * 38.9\frac{\mathrm{MJ}}{\mathrm{kg}} + 0.011kg * 17.2\frac{\mathrm{MJ}}{\mathrm{kg}} + 0.550kg * 18\frac{\mathrm{MJ}}{\mathrm{kg}} - 0.048kg * -2.441\frac{\mathrm{MJ}}{\mathrm{kg}}$$

$$H_u = 18.12\ MJ/kg$$

$$H_{u,wet} = 0.0641kg * 23\frac{\mathrm{MJ}}{\mathrm{kg}} + 0.0217kg * 38.9\frac{\mathrm{MJ}}{\mathrm{kg}} + 0.0032kg * 17.2\frac{\mathrm{MJ}}{\mathrm{kg}} + 0.161kg * 18\frac{\mathrm{MJ}}{\mathrm{kg}} - 0.750kg * -2.441\frac{\mathrm{MJ}}{\mathrm{kg}}$$

$$H_u = 7.10\ MJ/kg$$

$$H_u = 0.243kg * 23\frac{\mathrm{MJ}}{\mathrm{kg}} + 0.006kg * 38.9\frac{\mathrm{MJ}}{\mathrm{kg}} + 0.289kg * 17.2\frac{\mathrm{MJ}}{\mathrm{kg}} + 0.387kg * 18\frac{\mathrm{MJ}}{\mathrm{kg}} - 0.075kg * -2.441\frac{\mathrm{MJ}}{\mathrm{kg}}$$

$$H_u = 17.94\ MJ/kg$$

The 18.12 MJ/kg lower heating value of dried BSG does align with results from Milew et al. [14] who concluded a lower heating value between of 20.05 MJ/kg.

The resulting amount of electricity and heat from incineration was calculated using the current Swiss average efficiency of electricity and heat recovery from municipal solid waste incineration plants. The electricity efficiency amounts to 17%, and the heat efficiency to 33.2% (BFE, 2023). The calculated heating values as well as the resulting electricity and heat production from the incineration of BSG and BR are shown in Table 5.

Table S5: Electricity and heat co-production based on the lower heat value in MJ/kg fresh mass.

	BSG dried	BSG wet	BR
lower heat value [MJ/kg fresh mass]	18.12	7.10	17.94
the electricity production [MJ/kg fresh mass]	3.08	1.21	3.05
heat production [MJ/kg fresh mass]	6.02	2.36	5.96

The life cycle inventories of the substituted products were approximated using existing datasets from ecoinvent. Produced electricity was assumed to substitute the Swiss consumer mix, while produced heat was assumed to substitute heat from natural gas. All input data and datasets used in this modelling are listed in Table 6.

Table S6: Input data for heat and electricity co-creation from incineration of 1kg BSG and BR and its substitution products. Datasets are from the ecoinvent database.

Scenario	FU	Input	dataset	amount	unit	comment
Incineration – heat	2.36 MJ	BSG	Brewer Spent Grain (BSG) wet – VALISS	1 (0)	kg	system expansion and therefore 0 impact
		Or				
	5.96 MJ	BR	Barley Rootlets (BR) - VALISS			
		transport	Transport, freight, lorry, diesel, unspecified {RER}| market for transport, freight, lorry, unspecified	0.01	tkm	assumed distance for side stream transportation of 10 km
		incineration	Biowaste {GLO}| treatment of biowaste, municipal incineration	1	kg	incineration of the 1kg side stream
Incineration - electricity	1.21 MJ	BSG	Brewer Spent Grain (BSG) wet – VALISS	1 (0)	kg	system expansion and therefore 0 impact
		Or				
	3.05 MJ	BR	Barley Rootlets (BR) - VALISS			
		transport	Transport, freight, lorry, diesel, unspecified {RER}| market for transport, freight, lorry, unspecified	0.01	tkm	assumed distance for side stream transportation of 10 km
		incineration	Biowaste {GLO}| treatment of biowaste, municipal incineration	1	kg	incineration of the 1kg side stream

Heat substitution	2.36 MJ<br><br>5.96 MJ	Heat swiss	Heat, central or small-scale, natural gas {CH}| market for	2.36<br><br>5.96	MJ
Electricity substitution	1.21 MJ<br><br>3.05 MJ	Swiss electricity mix	Electricity, medium voltage {CH}| market for	1.21<br><br>3.05	MJ

1.3 Utilization Scenario 2 – anaerobic digestion of BSG and BR

The modeling of anaerobic digestion of the by-products is based on the approach by Beretta et al. [15]. For the life cycle inventory, an existing dataset from the ecoinvent database was used for all by-products: *Biowaste {CH}| treatment of biowaste by anaerobic digestion.* No allocation was made between waste treatment and biogas production; instead, system expansion was applied. The newly modelled biogas data set was then included and replaced the biogas dataset in the ecoivent dataset *Heat, central or small-scale, other than natural gas {CH}| heat and power co-generation, biogas, gas engine.* Therefore, the biogas production and its combustion are included.

Transport of the by-products to the biogas plant was modeled with an average distance of 10 km. The amount of biogas produced was calculated based on the specific biogas yield of each by-product. The energy content of biogas is 22.73 MJ/Nm³, assuming a methane content of 63.3%.[16] The specific biogas yield was therefore adjusted according to the actual methane content of each stream. Using the parameters in Table 7, following formulas were used:

$$S_{biogas} = Y_{biogas_{corr}} * E_{biogas}$$

$$Y_{biogas_{corr}} = Y_{biogas} / 0.633 * methane\ content$$

Table S7: Calculation of the specific biogas yield of BSG and BR.

	**1 kg BSG**	**1 kg BR**
moisture	75% water<br>25% DM	7.52% water<br>92.48% DM
methane content	58% [17]	
E_{biogas} = energy content (lower heating value) of biogas	22.73 MJ/Nm³ based on a methane content of 63.3% (Stucki, Jungbluth, & Leuenberger, 2011).	
Y_{biogas} = reported yields [Nm^3/kg DM] based on literature	0.5 Nm^3/kg oDM [17]<br>BSG generally has 0.95 oDM/DM ratio → 0.475 Nm^3/kg DM	Same assumptions as for BSG (since similar properties)
Y_{biogas_corr} = methan-corrected yield of biogas [Nm^3/kg DM]	0.475/0.633*0.58 = 0.435 Nm^3/kg DM	0.475/0.633*0.58 = 0.435 Nm^3/kg DM
$Y_{biogas\_corr\ of}$ 1kg product	0.435 Nm^3/kg DM *0.25<br>= 0.109 Nm^3/kg wet BSG	0.435 Nm^3/kg DM * 0.925<br>= 0.402 Nm^3/kg BR
S_{biogas} = specific biogas yield [MJ/kg DM]	0.109 Nm^3/kg BSG* 22.73 = 2.47 [MJ/kg wet BSG]	0.402 Nm^3/kg DM * 22.73 = 9.14 [MJ/kg BR]

Table S8: Inventory data for the utilization scenario anaerobic digestion of anaerobic digestion of 1kg BSG or BR and its resulting energy in form of heat and electricity. Datasets are from the ecoinvent database.

**input**	**calculation/comment/ dataset**	**heat BSG**	**heat BR**	**electricity BSG**	**electricity BR**

functional unit		*2.47*0.55 = 1.36 MJ*	*9.14*0.55 = 5.03 MJ*	*2.47*0.32= 0.79 MJ*	*9.14*0.32 = 2.92 MJ*
transport	0.001t*10 km = 0.01 tkm	0.01 tkm	0.01 tkm	0.01 tkm	0.01 tkm
anaerobic digestion	Disposal, biowaste, to anaerobic digestion, economic allocation {CH} U & Biogas, from biowaste, at storage, economic allocation {CH} U.	1 kg	1 kg	1 kg	1 kg

Biogas generated from BSG and BR was substituted accordingly. The reference products used for substitution are the average Swiss electricity mix for electricity and natural gas-based heat for thermal energy. For heat the dataset of ecoinvent is used: *Heat, central or small-scale, natural gas {CH}| market for heat, central or small-scale, natural gas*. And for electricity: *Electricity, low voltage {CH} | treatment of biogas, burned in micro gas turbine 100kWe* was used. Electrical efficiency is 0.32 and thermal efficiency 0.55 (Table 8).

1.3 Utilization Scenario 3 – feed of BSG and BR

For the feed scenario, BSG and BR are modelled as feed ingredients. A transport distance of 10 km is assumed, reflecting local utilisation of the side streams. Substitution is modelled on a nutrient-equivalent basis: 1 kg of BSG or BR feed is assumed to replace a compound feed mixture with comparable proximate composition.

BSG is primarily used as animal feed, either fresh or ensiled. Drying is mainly applied when ensiling is not feasible (e.g., regulatory restrictions) or when longer transport distances make frequent collection of fresh BSG impractical. Drying has little influence on the composition or nutritive value of the dry matter. Although BSG can be included in compound feed, this is limited; it is predominantly used as a single feedstuff. When incorporated into industrial feed formulations, it must be in dried form, and some manufacturers (e.g., UFA) do not use BSG at all. Overall, BSG is characterised as a protein-rich but energy-poor feed ingredient.

A nutrient-based substitute feed mix for 1 kg of BSG was therefore calculated to match its proximate composition (Table 9). The resulting formulation is presented in Table 10. No specific information on substitution of BR in animal feed could be identified; given its similar nutritional characteristics, the same substitute feed composition is applied for BR.

*Table S9: Input data for substitution feed mix for 1 kg of BSG and BR used as feed. * Proti-Grain® is a branded form of distillers dried grains with solubles (DDGS), a protein-rich by-product of ethanol production that is used as a feed ingredient for livestock.[18]*

input	dataset	amount for BSG	amount for BR	unit
wheat bran	Wheat bran, IP, at industrial mill/CH U [10]	120	120	g
Proti-grain DDGS*	DDGS, dehydrated, from wheat distillation, animal feed, at plant/FR U [19]	330	330	g
sunflower cake	Sunflower meal IP, at oil mill/CH U [10]	550	550	g

The feed scenarios for BSG and BR include transport of the raw materials to the pellet-production facility, pellet production itself, and subsequent transport of the pellets from the production site to retail. Electricity consumption for pelletizing and heat demand for drying are based on data from a study on greenhouse gas emissions of feed production and utilization.[20] The same processing steps are applied to the substitution feed mix, using the corresponding input materials listed in Table 10. All input data and ecoinvent datasets used in this modelling are summarized in Table 10.

Table S10: input data and its used datasets for the modelling of scenario feed and its substitution. Datasets are from the ecoinvent database.

scenario	FU	input	dataset	amount	unit	comment/source
Feed BSG	1kg	Feed material	Brewer Spent Grain (BSG) wet – VALISS<br>Barley Rootlets (BR) - VALISS	1 (0)	kg	0kg as system expansion
		Transport of side stream	Transport, freight, lorry, diesel, unspecified {RER}| market for	0.01	tkm	assumed distance for side stream transportation of 10 km
		Processing electricity – pressing in pellets	Electricity, low voltage {CH}| market for electricity, low voltage	0.315	MJ	Vellinga, T. V., Blonk, H., Marinussen, M., Zeist, W. J. Van,

		Processing heat - drying	Heat, central or small-scale, natural gas {CH}| market for	0.135	MJ	Boer, I. J. M. De, & Starmans, D. (2013). Methodology used in feedprint: a tool quantifying greenhouse gas emissions of feed production and utilization.
		Transport from feed production to animals	Transport, freight, lorry, diesel, unspecified {RER}| market for	0.07	tkm	assumed distance for side stream transportation of 70 km
Feed substitution	1kg	Feed material	See Table 9			
		Transport of side stream	Transport, freight, lorry, diesel, unspecified {RER}| market for transport, freight, lorry, unspecified	0.01	tkm	assumed distance for side stream transportation of 10 km
		Processing electricity – pressing in pellets	Electricity, low voltage {CH}| market for electricity, low voltage	0.315	MJ	Vellinga, T. V., Blonk, H., Marinussen, M., Zeist, W. J. Van, Boer, I. J. M. De, & Starmans, D. (2013).
		Processing heat - drying	Heat, central or small-scale, natural gas {CH}| market for heat, central or small-scale, natural gas	0.135	MJ	
		Transport from feed production to animals	Transport, freight, lorry, diesel, unspecified {RER}| market for transport, freight, lorry, unspecified	0.07	tkm	assumed distance for side stream transportation of 70 km

1.4 Utilization Scenario 4 – composting with BSG and BR

Composting is assessed as an additional valorization pathway. Composting yields a soil amendment that can substitute synthetic fertilizers. The quantity of resulting compost is adjusted to account for differences in dry-matter content relative to average compost. Nutrient substitution is modelled on a 1:1 basis, assuming that the compost replaces a synthetic fertilizer mix with an equivalent nutrient content.

Residues from the brewing industry are rich in plant nutrients, particularly phosphorus and potassium, and can therefore serve as a nutrient source for crops.[21] Field trials in south-western Nigeria showed that applying spent grain at 12.5 t/ha produced fruit yields comparable to those achieved with 200 kg/ha of NPK fertilizer.[22] NPK content of BSG and BR is estimated using literature values, as summarized in Table 11.

Table S11: Phosphate, nitrogen, and potassium content within 1 kg of dry matter of BSG and BR.

fertilizer	BSG	BR
N - NH_4^+ and NO_3^-	N: 2-5% [23] → 20-50g/kg DM<br>N 3.5% [24] → 35g/kg DM →<br><br>**3.5 g N = 15.5 g NO_3^-**	3.76 g/kg DM [25]<br><br>**16.7 g NO_3^-**
P – P_2O_5	0.293%--> 2.93 g/kg DM [26]<br>5.3g P/kg DM [27]<br><br>**5.3g P = 12.1g P_2O_5**	6.1g/ kg DM [25]<br><br>**14.0 g P_2O_5**
K – K_2O	2.9 g K/kg DM [25]<br>potassium levels (792–916 μg/g) → 0.916g/kg [24]<br>4.6g/kg DM [27]<br><br>**2.9 g K = 3.5 g K_2O**	17.5 g/kg DM [25]<br><br>**21.1 g K_2O**

Modelling of composting: On average, 0.5 kg of compost (fresh mass) is generated per kilogram of input biomass, assuming a mean compost dry-matter content of 39%.[28] To account for differences in dry-matter content between the modelled biomass and the reference compost, the compost yield is corrected using the following equation: corrected compost yield = 0.5 × (dry matter content of biomass / 0.39). Table 12 reports the dry-matter content used for each material and the resulting corrected compost yields.

Table S12: Dry matter and corrected compost yield of BSG and BR.

	BSG	BR
Dry matter content	25%	92.48%
Corrected compost yield [kg DM-eq]	0.5 × (0.25 / 0.39) = 0.321	0.5 × (0.9248 / 0.39) = 1.19

The data and ecoinvent datasets used for modelling the composting of 1 kg BSG and 1 kg BR are listed in Table 13. Based on the corrected compost yields, the resulting compost quantities are 0.321 kg for BSG and 1.19 kg for BR.

Table S13: input data used for modelling composting of BSG and BR. Datasets are from the ecoinvent database.

Input	Amount		Unit	dataset
	BSG	BR		
composting	1	1	kg	Biowaste, treatment of biowaste, industrial composting.

transport	0.01	0.01	tkm	Transport, freight, lorry, diesel, unspecified {RER}| market for transport, freight, lorry, unspecified

The life cycle inventories of the substituted products were approximated using existing inventories from the ecoinvent database (Table 14). Synthetic fertilizers were approximated with inventories of mineral N, P_2O_5, and K_2O fertilizers, respectively.

Table S14: Input data used for modelling the substitution products (synthetic NPK fertilizer) of BSG- and BR-compost. Datasets are from the ecoinvent database.

**input**	**amount**		**unit**	**dataset**
	BSG	BR		
P-fertilizer	0.012 * 0.25 = 0.003	0.014 * 0.9248 = 0.013	kg	Inorganic phosphorus fertiliser, as P2O5 {CH}| market for inorganic phosphorus fertiliser, as P2O5
K-fertilizer	0.0035 * 0.25 = 0.000875	0.021 * 0.9248 = 0.019	kg	Inorganic potassium fertiliser, as K2O {CH}| market for inorganic potassium fertiliser, as K2O
N-fertilizer	0.015 * 0.25 = 0.00375	0.016 * 0.9248 = 0.015	kg	Inorganic nitrogen fertiliser, as N {CH}| market for inorganic nitrogen fertiliser, as N

2. Economic Allocation

Economic allocation was applied to the co-products BSG and BRL (Table 15). The resulting allocation factors for beer production are: BRL 0.02%, BSG 0.22%, and beer 99.76%.

Table S15: Economic allocation calculation of BSG and BRL.

**products from beer production**		**economic value**
beer	13l = 13.3 kg	1.5 CHF/kg [29]
BR	0.04 kg [30]	0.075 CHF/kg [31,32]
BSG wet	1.3 kg [33]	0.035 CHF/kg [34]

**value added**	
beer	13.3kg *1.5 CHF = 19.95 CHF
BR	0.04 kg * 0.075 CHF = 0.003 CHF
BSG	1.3 kg * 0.035 CHF = 0.0455 CHF

**allocation factor**	
beer	19.95/19.9985 = **99.76%**
BR	0.003/19.9985 = **00.02%**
BSG	0.0455/19.9985 = **00.22%**

3. Results

3.1. Results of extrudates with economic allocation

The results in CO_2-eq (Table 16) and eco-points (Table 17) for the more detailed production stages of extrudates, including ingredients, side stream, HMEC, and packaging & storage, are presented below.

Table S16: Results in CO_2-eq of extrudates per production stage based on the IPCC2021 GWP100 method. PPI_160_EU is a scenario calculated with an average European electricity mix.

scenario	ingredients	side stream	HMEC	packaging & storage	sum
PPI_160	0.881	0	0.0124	0.183	1.08
BSG1_160	0.812	0.0002	0.0126	0.183	1.01
BSG2_160	0.735	0.0003	0.0128	0.183	0.94
BR1_160	0.812	0.0005	0.0126	0.183	1.01
BR2_160	0.713	0.001	0.0124	0.183	0.91
PPI_160_EU	0.881	0	0.18	0.183	1.25

Table S17: Results in eco-points of extrudates per production stage based on the Ecological Scarcity Method. PPI_160_EU is a scenario calculated with an average European electricity mix.

scenario	ingredients	side stream	HMEC	packaging & storage	sum
PPI_160	11’715	0	87	335	12’137
BSG1_160	10’767	0	88	335	11’206
BSG2_160	9’720	1	89	335	10’177
BR1_160	10’767	1	88	335	11’218
BR2_160	9’420	3	87	335	9’847
PPI_160_EU	11’715	0	354	335	12’404

3.2. Results of all scenarios with avoided burden, system expansion

Table 18 shows the CO_2-eq of the utilized scenarios of 1kg BR and table 19 the CO_2-eq of the utilized scenarios of 1kg BSG, their reference products which are substituted and the resulting net benefit.

Table S18: Results in kg CO_2-eq of all scenarios including substitution products based on system expansion approach per kg of BR valorized or utilized.

scenario	Amount	utilized product [CO_2-eq]	amount	reference product [CO_2-eq]	net benefit [CO_2-eq]
extrudates BR 1	25.1 kg	25.3	25.1 kg	27.0	-1.64
extrudates BR 2	12.8 kg	11.6	12.8 kg	13.8	-2.14
feed	1 kg	0.0240	1 kg	0.413	-0.389
fertilizer	1.19 kg	0.0510	1.19 kg	0.195	-0.144
incineration	9.01 MJ	0.0416	9.01 MJ	0.367	-0.325
anaerobic digestion	7.95 MJ	0.0381	7.95 MJ	0.373	-0.335

Table S19: Results in kg CO_2-eq of all scenarios including substitution products based on system expansion approach per kg of BSG valorized or utilized.

scenario	amount	utilized product [CO_2-eq]	amount	reference product [CO_2-eq]	net benefit [CO_2-eq]
extrudates BSG 1	25.1 kg	25.4	25.1 kg	27.0	-1.67
extrudates BSG 2	12.4 kg	11.6	12.4 kg	13.3	-1.74
feed	1 kg	0.0241	1 kg	0.413	-0.388
fertilizer	0.321 kg	0.0510	0.321 kg	0.0368	0.0142
incineration	3.57 MJ	0.0416	3.57 MJ	0.101	-0.0597
anaerobic digestion	2.15 MJ	0.0336	2.15 MJ	0.101	-0.0673

3.3. Visualisation of Electricity Scenario

The high-moisture extrusion cooking (HMEC) stage contributes 0.012 kg CO_2-eq per kg extrudate, corresponding to ~1.3% of the total impacts of the four side stream recipes (PPI_160). This is substantially lower than the ~20% extrusion contribution reported in the study on plant based meats produced by high-moisture extrusion.[6] The discrepancy is primarily driven by the electricity mix. Replacing the Swiss electricity mix, used in our assessment, with a European mix (ENTSO-E) increases the HMEC contribution from ~1% to ~15% (Figure 1).

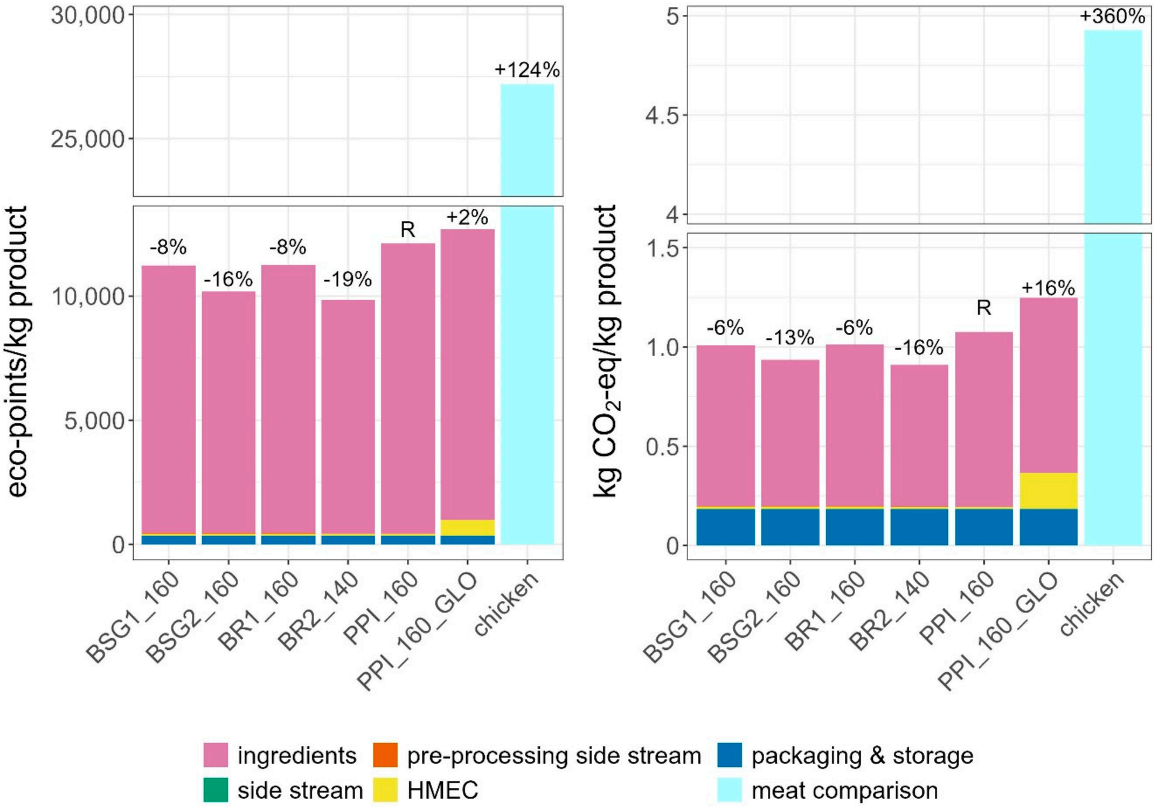

Figure S2: Overall environmental impacts (eco-points, left) and life cycle greenhouse gas emissions (kg CO_2-eq, right) of 1 kg product with partial substitution of pea protein by BR and BSG, assessed using the Ecological Scarcity Method and the IPCC 2021 methodology. Ingredients: production of pea protein and other ingredients, side stream: BR & BSG economic allocation, pre-processing side stream: pre-processing including drying & milling for the scenarios, HMEC: high moisture extrusion cooling, packaging & storage: material and cooled storage. PPI_160 (R): 100% Pea protein isolate at 160 °C, BSG1_160: 10% Brewers spent grain at 160 °C, BSG2_160: 20% Brewers spent grain at 160 °C, BR1_160: 10% Barley rootlets at 160 °C, BR2_140: 20% Barley rootlets at 140 °C. PPI_160_GLO: scenario with European electricity mix.

4. Literature

(1) Amienyo, D. *Life Cycle Sustainability Assessment in the UK Beverage Sector*; The University of Manchester, 2012. https://research.manchester.ac.uk/en/studentTheses/life-cycle-sustainability-assessment-in-the-uk-beverage-sector/ (accessed 2025-11-19).

(2) Fabani, M.; Capossio, J.; Reyes-Urrutia, A.; Rodriguez, R.; Mazza, G. Sustainable Natural Solar Drying of Microbreweries Spent Grains: A Comparison with Common Electric Convective Drying. *IOP Conf. Ser. Earth Environ. Sci.* **2022**, *952* (1), 012004. https://doi.org/10.1088/1755-1315/952/1/012004.

(3) Sobulska, M.; Wawrzyniak, P.; Woo, M. W. Superheated Steam Spray Drying as an Energy-Saving Drying Technique: A Review. *Energies* **2022**, *15* (22), 8546. https://doi.org/10.3390/en15228546.

(4) Neylon, E.; Arendt, E. K.; Lynch, K. M.; Zannini, E.; Bazzoli, P.; Monin, T.; Sahin, A. W. Rootlets, a Malting By-Product with Great Potential. *Fermentation* **2020**, *6* (4), 117. https://doi.org/10.3390/fermentation6040117.

(5) Naibaho, J.; Korzeniowska, M. The Variability of Physico-Chemical Properties of Brewery Spent Grain from 8 Different Breweries. *Heliyon* **2021**, *7* (3), e06583. https://doi.org/10.1016/j.heliyon.2021.e06583.

(6) Bonales, J.; Barrera-Ramirez, J.; Earthshift Global; Sojo, A.; Earthshift Global; Ayer, N.; Earthshift Global. *Comparative Life Cycle Assessment of Plant-Based Meats and Conventional Animal Meats*; The Good Food Institute, 2024. https://doi.org/10.62468/casv3213.

(7) Retsch. *Schlagrotormühle SR 300*. Schlagrotormühle SR 300. https://www.retsch.de/de/produkte/zerkleinern/rotormuehlen/sr-300/ (accessed 2025-11-06).

(8) Baker Perkins. *High Moisture Meat Analogues (HMMA)*. Food Extrusion. https://www.bakerperkins.com/food-extrusion/products/high-moisture-meat-analogues/ (accessed 2025-11-19).

(9) UN, U. N. E. *Single-Use Supermarket Food Packaging and Its Alternatives: Recommendations from Life Cycle Assessments*; 2022.

(10) ZHAW. *Agri-food Database, www.zhaw.ch/IUNR/agri-food*; ZHAW Institute for Natural Resource Sciences: Wädenswil, 2025. https://www.zhaw.ch/de/forschung/projekt/71134.

(11) Blonk Agrifootprint. *Agri-Footprint - Part 2 - Description of data - Version 1.0*. doczz.net. https://doczz.net/doc/7635016/agri-footprint-2.0-part-2--description-of-data (accessed 2025-11-10).

(12) Broekema, R.; Smale, E. *Nulmeting Peulvruchten*; Gouda, Netherlands, 2011. https://adoc.pub/nulmeting-peulvruchten.html (accessed 2025-11-10).

(13) Van Veghel, A. *The environmental impact of green proteins and their role in a healthy diet*. https://blonksustainability.nl/news/revealing-the-environmental-impact-of-plant-proteins?utm_source=chatgpt.com#gsc.tab=0 (accessed 2025-11-10).

(14) Milew, K.; Manke, S.; Grimm, S. L.; Haseneder, R.; Herdegen, V.; Braeuer, A. Application, Characterisation and Economic Assessment of Brewers' Spent Grain and Liquor. *J. Inst. Brew.* **2022**, *128* (3). https://doi.org/10.1002/jib.697.

(15) Beretta, C.; Stucki, M.; Hellweg, S. Environmental Impacts and Hotspots of Food Losses: Value Chain Analysis of Swiss Food Consumption. *Environ. Sci. Technol.* **2017**, *51* (19), 11165–11173. https://doi.org/10.1021/acs.est.6b06179.

(16) Stucki, M.; Jungbluth, N.; Leuenberger, M. *Life Cycle Assessment of Biogas Production from Different Substrates*; ESU-services GmbH: Uster, 2011. https://esu-services.ch/fileadmin/download/stucki-2011-LCA_Biogas_v1.0.pdf.

(17) Weger, A.; Stenzel, F.; Hornung, A. *Optimized Fermentation Concept for Brewer's Spent Grain*; 23rd European Biomass Conference and Exhibition; Sulzbach-Rosenberg, Austria, 2015. https://www.umsicht-suro.fraunhofer.de/content/dam/umsicht-suro/de/documents/Publikationen/2017/EUBCE/Weger_EUBCE15_PAPER.pdf.

(18) ProtiGrain. *ProtiGrain® - Das Plus an Protein | CropEnergies AG*. https://www.cropenergies.com/de/produkte/lebens-und-futtermittel/protigrain (accessed 2025-10-02).
(19) ADEME. *Base de Données Agribalyse v3.1*; 2022. https://agribalyse.ademe.fr.
(20) Vellinga, T. V.; Blonk, H.; Marinussen, M.; Zeist, W. J. van; Starmans, D. a. J. Methodology Used in FeedPrint: A Tool Quantifying Greenhouse Gas Emissions of Feed Production and Utilization. **2013**.
(21) Alayu, E.; Leta, S. Brewery Sludge Quality, Agronomic Importance and Its Short-Term Residual Effect on Soil Properties. *Int. J. Environ. Sci. Technol.* **2020**, *17* (4), 2337–2348. https://doi.org/10.1007/s13762-020-02630-2.
(22) Ojeniyi, S. O.; Awodun, M. A.; Odedina, S. A. Effect of Animal Manure Amended Spent Grain and Cocoa Husk on Nutrient Status, Growth and Yied of Tomato. *ResearchGate* **2007**, *2* (4), 406–410.
(23) Jackowski, M.; Niedźwiecki, Ł.; Jagiełło, K.; Uchańska, O.; Trusek, A. Brewer's Spent Grains—Valuable Beer Industry By-Product. *Biomolecules* **2020**, *10* (12), 1669. https://doi.org/10.3390/biom10121669.
(24) García, D. C.; Villalba, I.; Savino, N.; Nazareno, M. A. Nutritional and Functional Characterization of Different Types of Brewer's Spent Grain Flours. *Food Biosci.* **2025**, *64*, 105890. https://doi.org/10.1016/j.fbio.2025.105890.
(25) Feedipedia. *Brewers grains, dehydrated | Feedipedia*. https://www.feedipedia.org/node/11893?utm_source=chatgpt.com (accessed 2025-10-02).
(26) Senthil, Murugan. S. Mineral Profiling of Brewer's Spent Grain. **2015**, *29*.
(27) Feedinamics. *Brewers grains, dried | Tables of composition and nutritional values of feed materials INRA CIRAD AFZ*. https://feedtables.com/content/brewers-grains-dried?utm_source=chatgpt.com (accessed 2025-10-02).
(28) Dinkel, F.; Zschokke, M.; Schleiss, K. *Ökobilanzen zur Biomasseverwertung*; Carbotech AG, 2012. https://carbotech.ch/cms/wp-content/uploads/LCA_Biomasseverwertung.pdf.
(29) Heineken. *Heineken N.V. reports on 2023 full year results*. Heineken N.V. reports on 2023 full year results. https://www.theheinekencompany.com/newsroom/heineken-nv-reports-on-2023-full-year-results/ (accessed 2025-11-19).
(30) Rani, H.; Bhardwaj, R. D. Quality Attributes for Barley Malt: "The Backbone of Beer." **2021**, *86* (8), 3322–3340. https://doi.org/10.1111/1750-3841.15858.
(31) European Comission. *European Commission | Prices dashboard*. Prices dashboard. https://agridata.ec.europa.eu/extensions/DashboardPrice/PricesDashboard.html (accessed 2025-09-29).
(32) Tridge. *Global Barley Malt Price*. Tridge. https://dir.tridge.com/prices/barley-malt (accessed 2025-09-29).
(33) Lynch, K. M.; Steffen, E. J.; Arendt, E. K. Brewers' Spent Grain: A Review with an Emphasis on Food and Health. *J. Inst. Brew.* **2016**, *122* (4), 553–568. https://doi.org/10.1002/jib.363.
(34) Baiano, A.; la Gatta, B.; Rutigliano, M.; Fiore, A. Functional Bread Produced in a Circular Economy Perspective: The Use of Brewers' Spent Grain. *Foods* **2023**, *12* (4), 834. https://doi.org/10.3390/foods12040834.
