# Supplementary figures and images for: Upcycling Brewer’s Spent Grain and Barley Rootlets by Partial Substitution of Pea Protein Isolate in Extruded High Moisture Meat Analogues
Source: Foods. 2026 Apr 10;15(8):1327. doi: 10.3390/foods15081327 (PMC13114482; doi:10.3390/foods15081327)

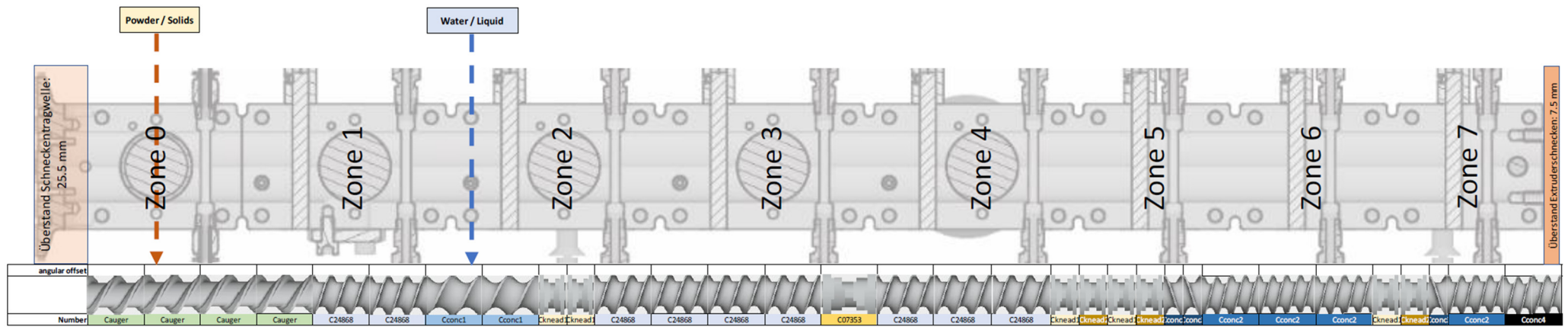

Figure S1: Extruder screw configuration.

Supplement: Supplementary file 1 [file foods-15-01327-s001.zip › SupplementaryMaterial S1.pdf]

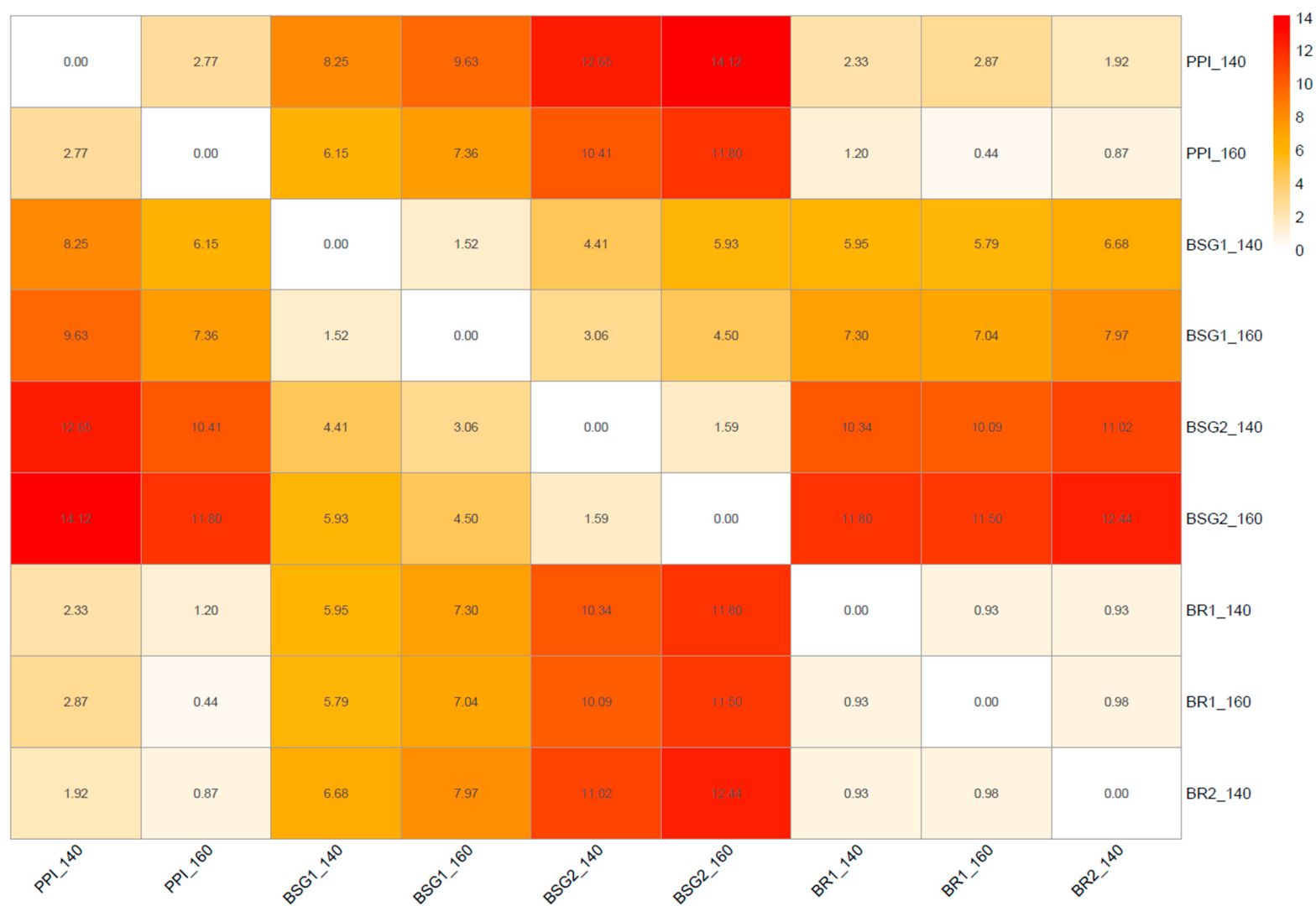

Figure S3: Delta E (CIELAB) values to show color difference between samples.

Supplement: Supplementary file 1 [file foods-15-01327-s001.zip › SupplementaryMaterial S3.pdf]
